# Cross-Species Analysis of ABA-Induced Phosphosignaling Landscapes in Rice, Soybean, and Arabidopsis

**DOI:** 10.3390/proteomes14010004

**Published:** 2026-01-20

**Authors:** Hinano Takase, Sotaro Katagiri, Takuma Ide, Aina Nagano, Haruki Sakurai, Hana Kokubo, Taiki Yanagisawa, Masanori Okamoto, Taishi Umezawa

**Affiliations:** 1 Graduate School of Bio-Applications and Systems Engineering, Tokyo University of Agriculture and Technology, Tokyo 184-8588, Japan; s255008q@st.go.tuat.ac.jp (H.T.); s231343y@st.me.tuat.ac.jp (T.I.); s245044z@st.me.tuat.ac.jp (A.N.); 2 RIKEN Center for Sustainable Resource Science (CSRS), Yokohama 230-0045, Japan; sotaro.katagiri@riken.jp (S.K.); okamo@riken.jp (M.O.); 3 Graduate School of Advanced Interdisciplinary Science, Tokyo University of Agriculture and Technology, Tokyo 183-8538, Japan; s256250y@st.me.tuat.ac.jp; 4 Faculty of Agriculture, Tokyo University of Agriculture and Technology, Tokyo 183-8538, Japan; s220009z@st.me.tuat.ac.jp (H.K.); s225189v@st.me.tuat.ac.jp (T.Y.)

**Keywords:** Arabidopsis, rice, soybean, phosphoproteome, abscisic acid, protein kinase

## Abstract

Background: Abscisic acid (ABA) is a key phytohormone that regulates plant growth and stress responses through protein phosphorylation. While ABA-induced phosphosignaling has been extensively studied in *Arabidopsis thaliana*, its conservation and divergence across plant species remain unclear. Methods: Here, we performed phosphoproteomic analysis using LC-MS/MS in Arabidopsis, rice (*Oryza sativa*), and soybean (*Glycine max*) to compare ABA-responsive phosphorylation profiles among monocots, dicots, and legumes. Results: ABA treatments on Arabidopsis, rice, and soybean seedlings yielded approximately 24,604, 18,865, and 24,930 phosphopeptides, respectively. Comparative analyses revealed both conserved and species-specific ABA-responsive phosphoproteins. Conclusions: This work provides insights into the evolutionary diversification of ABA signaling and its potential applications in improving crop stress tolerance.

## 1. Introduction

Abscisic acid (ABA) is a major phytohormone that orchestrates plant growth, development, and adaptive responses to various abiotic stresses, including drought and high salinity. Under osmotic stress, ABA rapidly induces stomatal closure to limit transpirational water loss, thereby contributing critically to the maintenance of plant water homeostasis [1]. During seed maturation, ABA accumulates to establish seed dormancy and suppress germination, ensuring that seedling emergence occurs under favorable environmental conditions [2,3].

Over the past decade, substantial progress has been made in elucidating the molecular basis of ABA signaling. The PYR/PYL/RCAR–PP2C–SnRK2 module is now recognized as the core signaling cascade that mediates ABA perception and downstream responses [4,5]. In *Arabidopsis thaliana*, subclass III SnRK2s—namely SnRK2.2, SnRK2.3, and SnRK2.6/OST1—play essential roles in ABA-activated signaling [6,7,8]. In the absence of ABA, clade A PP2Cs repress SnRK2 activity through dephosphorylation, whereas ABA-bound PYR/PYL/RCAR receptors inhibit PP2Cs, releasing SnRK2s to phosphorylate transcription factors, ion channels, and other signaling components that mediate physiological ABA responses [9,10,11].

Comparative analyses have further revealed that the architecture of the ABA core signaling network is evolutionarily well conserved across land plants. Key components—including PYR/PYL receptors, clade A PP2Cs, SnRK2 kinases, and ABF/ABI5-type basic leucine zipper (bZIP) transcription factors—are present in bryophytes, monocotyledonous crops such as rice and maize, and many dicotyledonous species [4,8]. Functional studies have demonstrated partial cross-species complementation, supporting the hypothesis that the core signaling logic is shared across taxa [6]. Moreover, structural and biochemical analyses of ABA receptor–PP2C interactions strongly suggest that the fundamental receptor-mediated inhibition mechanism predates the diversification of seed plants [12]. Nonetheless, the extent to which ABA-induced downstream phosphorylation events are conserved remains unclear, as only limited information is available regarding the dynamics of post-translational regulation in crop species [13].

While large-scale phosphoproteomic studies have begun to reveal evolutionary patterns in phosphorylation site conservation [14,15], these analyses have primarily focused on basal phosphorylation states rather than hormone-induced dynamics. Thus, the degree to which ABA-triggered phosphorylation responses are preserved across phylogenetically distant species—particularly between monocots and eudicots—remains largely unexplored. Moreover, existing studies rarely integrate a legume crop, leaving a substantial gap in understanding lineage-specific ABA signaling evolution. Soybean (*Glycine max*), a major legume crop with a distinct evolutionary trajectory relative to both Arabidopsis and rice, provides a valuable system to examine diversification of ABA-responsive phosphoregulation.

Addressing these limitations is essential for three reasons: (1) to uncover universal stress-adaptation mechanisms that are shared across angiosperms; (2) to identify species- or lineage-specific phosphorylation signatures that may underlie differential stress tolerance among crops; (3) to reveal evolutionary constraints and innovations within ABA signaling networks at the post-translational level.

In this study, we performed a comprehensive label-free MS-based phosphoproteomic analysis of ABA-treated seedlings of *Arabidopsis thaliana*, *Oryza sativa* (rice), and *Glycine max* (soybean). By integrating ortholog mapping, phosphorylation site annotation, and quantitative dynamics across three phylogenetically distant species, we generated one of the largest comparative datasets of ABA-responsive phosphorylation to date. This framework allowed us to dissect conserved and lineage-specific features of ABA-induced phosphoregulation, thereby providing new insights into the evolutionary logic and diversification of plant stress signaling networks.

## 2. Materials and Methods

### 2.1. Plant Materials and Growth Conditions

*Arabidopsis thaliana* ecotype Columbia (Col-0) was used for this study. Seeds were sterilized and sown on 0.8% (*w*/*v*) germination agar medium (GM) containing 1% (*w*/*v*) sucrose. After vernalization at 4 °C in the dark for 4 days, they were grown under a 16 h/8 h (light/dark) photoperiod of 90 µmol m^−2^ s^−1^ photon flux density (LED white lamps) at 22 °C for 2 weeks. 50 μm ABA was absorbed from the roots, and the tissues were harvested after 0, 15, 30 and 90 min. The samples were then stored at −80 °C.

*Oryza sativa* L. cv. Nipponbare and *Glycine max* (L.) Merrill were used for this study. Seeeds were soaked in water at room temperature for 24 h before sowing in soil. Plants were grown under a 16 h/8 h (light/dark) photoperiod of 150 µmol m^−2^ s^−1^ photon flux density (LED white lamps) at 22 °C for 2 weeks. 2-week-old plants were immersed in Hoagland hydroponic solution and grown for an additional 1 week. 50 μm ABA was absorbed from the roots, and the tissues were harvested after 0, 15, 30 and 90 min. The samples were then stored at −80 °C.

### 2.2. In-Gel Phosphorylation Assays

In-gel phosphorylation assay was performed as previously described with slight modifications [16]. Crude extracts were obtained by adding extraction buffer (20 mM HEPES-KOH (pH 7.5), 0.5% TritonX-100, 100 mM NaCl, 5 mM MgCl_2_, 0.1 mM EDTA, 1 mM Na_3_VO_4_, 25 mM NaF, 50 mM β-glycerophosphate, 20% (*v*/*v*) glycerol, and 1% (*v*/*v*) protease inhibitor cocktail (Sigma-Aldrich, St. Louis, MO, USA)); protein concentration was measured using the Bradford method. A total of 20 μg protein from each sample was used for the phosphorylation assay. This assay is presented with three biologically independent samples.

### 2.3. RNA Extraction and RT-qPCR Analysis

Total RNA was extracted as described previously, and 500 ng of total RNA was used for reverse transcription using ReverTra Ace qPCR RT Master Mix with gDNA Remover (TOYOBO, Osaka, Japan) [16]. RT-qPCR analysis was performed using THUNDERBIRD^®^ SYBR™ qPCR Mix (TOYOBO) with Light Cycler 96 (Roche Life Science, Basel, Switzerland). Each transcript was normalized by GAPDH (*Arabidopsis thaliana*), OsRUBQ2 (*Oryza sativa*), GmTubulin (*Glycine max*) as previously described [17,18]. Data are presented as means ± SE (*n* = 3 biologically independent samples). The gene-specific primers used for RT-qPCR are listed in Supplementary Data S7.

### 2.4. Sample Preparation for Phosphoproteomic Analysis

Three biologically independent replicates were conducted for each plant in phosphoproteomic analysis. Protein extraction and digestion were performed according to a previous study with some modifications [16]. In brief, total protein lysates were extracted from plants in protein extraction buffer [100 mM Tris-HCl (pH 9.0), 6 M guanidine hydrochloride (Gdn-HCl)], followed by heating the lysates for 5 min at 95 °C. Place the lysate samples on ice, sonicate the samples using a micro ultrasonic homogenizer (Q125, QSONICA, Newtown, CT, USA), and centrifuge at 17,400× *g* for 20 min at 4 °C. The supernatant was then precipitated by the methanol-chloroform method, and the protein pellets were resuspended in a digestion buffer containing 100 mM Tris-HCl (pH 9.0), 12 mM SLS, and 12 mM SDC. 400 μg of protein per sample was incubated in a reduction buffer [10 mM DTT, 100 mM ammonium bicarbonate] for 30 min at 24 °C, followed by alkylation buffer [40 mM IAM, 100 mM ammonium bicarbonate] for 30 min at 24 °C in the dark. After a five-fold dilution with 100 mM ammonium bicarbonate, proteins were digested overnight at 37 °C with 2 µg trypsin (Promega, Madison, WI, USA). Phase transfer surfactants (such as SLS, SDC) were removed according to a previous study 58. After removal, 10% of the total volume of peptide samples was set aside for global proteomic analysis, and phosphopeptides were enriched using hydroxy acid-modified metal oxide chromatography (HAMMOC) as described in previous studies [19]. Digested peptides or enriched phosphopeptides were desalted using an in-house Stage-tip made with SDB Empore disks (CDS). After desalting, the phosphopeptides were dried and stored at −80 °C until LC-MS/MS analysis.

### 2.5. LC-MS/MS Analysis and Raw Data Processing

Prepared peptide samples were analyzed using a nano LC system, Easy-nLC 1200 (Thermo Scientific, Waltham, MA, USA), connected in line with a quadrupole-Orbitrap mass spectrometer, Orbitrap Exploris 480 (Thermo Scientific), equipped with an aerodynamic high-field asymmetric waveform ion mobility spectrometry (FAIMS) device, FAIMS Pro (Thermo Scientific).

Dried peptides were resuspended in a solution of 2% (*v*/*v*) acetonitrile (ACN) with 0.1% (*v*/*v*) formic acid (FA) and then loaded directly into a C18 nano HPLC capillary column (NTCC-360/75-3, 75 µm ID × 15 cm L, Nikkyo Technos, Tokyo, Japan). Peptides were eluted at 300 nL/min, at 60 °C with nonlinear gradient program for 140 min for phosphoproteomic analysis. The 140 min program was executed under the following conditions: 0–5 min, B 6% held; 5–79 min, B 6–23%; 79–107 min, B 23–35%; 107–125 min, B 35–50%; 125–130 min, B 50–90%; 130–140 min, B 90% held. Eluted peptides were ionized at a source voltage of 2.2 kV and detected using data-dependent acquisition (DDA) in positive ion mode. MS1 spectra were collected in the range of 375–1500 *m*/*z*. The resolving power was set to 60,000. MS2 spectra were collected in the range above 120 *m*/*z* at a resolving power of 30,000. In the FAIMS compensation voltage (CV), −40/−60 CV conditions were used, and the resolution was set to “Standard” (inner temperature (IT) 100 °C/outer temperature (OT) 100 °C) in both conditions.

Peptide/protein identification and MS1-based label-free quantification (LFQ) were performed using Proteome Discoverer 2.5 (PD2.5) (Thermo Scientific). MS/MS spectra were searched using SEQUEST HT (PD2.5) algorithms. The following search parameters were used as follows: [Digestion Enzyme; trypsin], [Maximum Missed Cleaves; 2], [Peptide Length; 6–144], [Precursor Mass Tolerance; 10 ppm], [Fragment Mass Tolerance; 0.02 Da], [Static Modification; cysteine (C) carbamidomethylation], [Variable Modification; methionine (M) oxidation/N-terminal acetylation/serin (S) threonine (T) tyrosine (Y) phosphorylation], [Maximum Variable Modifications; 3]. Peptide validation was performed using the Percolator algorithm, and only high confidence peptides (false discovery rate (FDR) < 1%) were used for protein inference and MS1-based LFQ.

### 2.6. Analysis of Proteomic Data

Unless otherwise noted, basic calculations related to proteomic data were performed in Excel program. GO enrichment analysis was performed as follows: the protein IDs in each Excel sheet (Supplementary Data S5) were uploaded to agriGO (https://systemsbiology.cau.edu.cn/agriGOv2/, accessed on 10 December 2025) or DAVID (https://davidbioinformatics.nih.gov/, accessed on 10 December 2025) program. The ortholog groups of *Oryza sativa* and *Arabidopsis thaliana* genes were obtained from the OlthoMCL database [20]. To classify *Glycine max* genes into orthologous groups, closest genes among registered in OrthoMCL were extracted using blastp search. The blastp search threshold was set to E value < 10^−5^. Genes of *Glycine max* were classified into orthologous groups same as the gene with lowest E value. Motif analysis was performed as follows: The phosphorylation sites of phosphopeptides detected by Proteome Discoverer were centered, and the 6 amino acids upstream and downstream were extracted as foreground sequences. As background sequences, the 6 amino acids upstream and downstream of serine, threonine, and tyrosine residues in all registered genes of the corresponding plant species were extracted. Motif creation utilized rmotifex and ggseqlogo package from the motifR package (https://github.com/wangshisheng/motifeR, accessed on 10 December 2025). The R script used is available at https://github.com/sou-06/Phospho_Logo_PD, accessed on 10 December 2025. Analyses were performed using R version 4.5.2.

## 3. Results and Discussion

Abscisic acid (ABA) is a key phytohormone that regulates agriculturally important traits, including seed dormancy, stomatal movement, and drought tolerance. Elucidating the mechanisms underlying ABA responses is therefore directly relevant to crop improvement. Under soil drying or osmotic stress, increases in endogenous ABA concentrations trigger a cascade of drought-associated responses, such as stomatal closure, accumulation of compatible solutes, and induction of LEA proteins [21]. Because many of these processes are controlled by protein phosphorylation, phosphoproteome profiling provides a powerful approach to dissect the signaling dynamics of ABA at a systems level.

In this study, we performed time-resolved phosphoproteomic analyses in *Arabidopsis thaliana*, rice, and soybean to characterize the temporal and evolutionary properties of ABA responses and to identify conserved and lineage-specific features among the three species. Our goal was to advance understanding of the phylogenetic conservation of ABA signaling and to uncover potential mechanisms unique to crop species.

### 3.1. SnRK2 Activation and ABA-Responsive Gene Expression

ABA effects are known to differ between organs. Generally, ABA inhibits shoot growth, whereas its effect on root elongation is concentration-dependent: high concentrations suppress growth, while lower concentrations promote it [22]. To accurately evaluate these organ-specific characteristics, we independently analyzed phosphoproteome dynamics in shoots and roots.

**Figure 1:**
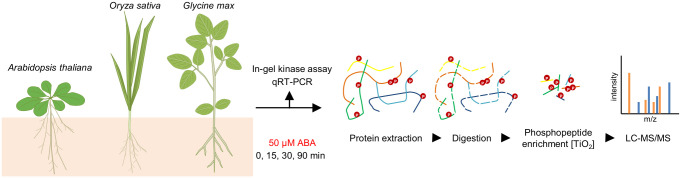
Schematic overview of the experimental workflow for the phosphoproteomic analysis. Plants were grown hydroponically and treated with 50 µM ABA. Root and shoot samples were collected at 0, 15, 30, and 90 min after treatment.

Seedlings of Arabidopsis, rice, and soybean were treated with 50 μM ABA, and shoot and root samples were collected at 0, 15, 30, and 90 min after treatment (Figure 1). To verify that the plants responded to ABA, we first examined SnRK2 activation and the induction of canonical ABA-responsive genes. SnRK2 activity was assessed by in-gel kinase assays using histone as a general substrate, a method widely used to evaluate ABA-activated SnRK2s in Arabidopsis [8,23] and also applicable to rice SAPK8/9/10 [24].

**Figure 2:**
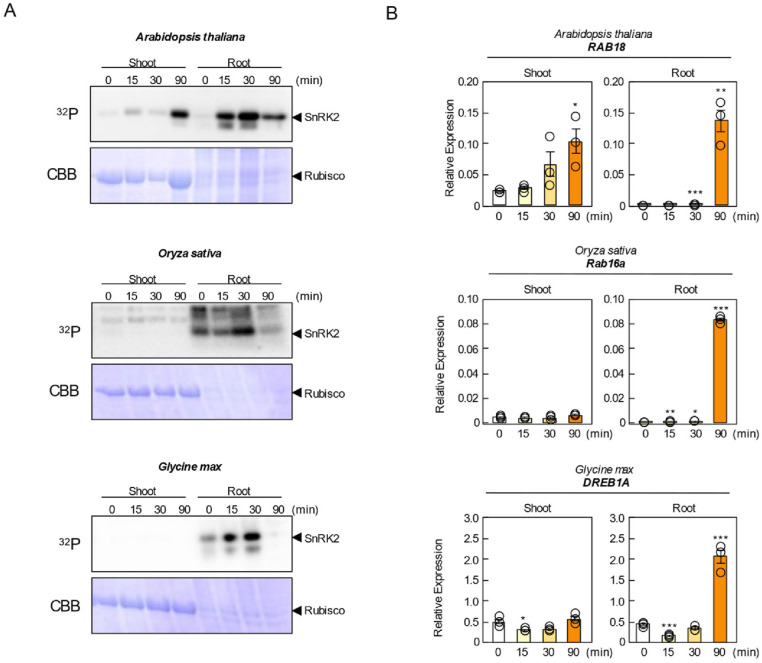
Sample evaluation of ABA responsiveness in Arabidopsis, rice, and soybean. (**A**) ABA-activated protein kinases were analyzed by an in-gel phosphorylation assay. Crude protein extracts were prepared from Arabidopsis, rice, and soybean seedlings treated with 50 µM ABA for 0, 15, 30, and 90 min. (**B**) Relative expression levels of *RAB18* (Arabidopsis), *Rab16a* (rice), and *DREB1A* (soybean) in response to ABA treatment at 0, 15, 30, and 90 min. Each transcript was normalized by GAPDH (Arabidopsis), OsRUBQ2 (rice), GmTubulin (soybean). Data are presented as means ± SE (*n* = 3 biologically independent samples). Asterisks indicate statistically significant differences as determined by Student’s *t*-test (* *p* < 0.05, ** *p* < 0.01, *** *p* < 0.001; not significant, *p* > 0.05).

In Arabidopsis, SnRK2 activation was detected in shoots at 15 min after ABA treatment and peaked at 90 min (Figure 2A). In roots, the kinase activity also increased by 15 min, peaked at 30 min, and declined by 90 min. In contrast, no ABA-induced SnRK2 activation was detected in the shoots of rice or soybean under our experimental conditions. However, in both species, root SnRK2 activity peaked at 30 min and decreased by 90 min (Figure 2A). One possible explanation for the reduced phosphorylation observed at 90 min is a decrease in endogenous ABA levels caused by ABA catabolism, as ABA-inducible CYP707A genes, encoding ABA 8′-hydroxylases, rapidly degrade ABA [25].

We next quantified ABA-responsive gene expression by qRT-PCR using Arabidopsis RAB18, rice Rab16a, and soybean GmDREB1A as marker genes [17,18,26]. RAB18 showed robust induction in both shoots and roots at 90 min, whereas Rab16a and GmDREB1A were markedly induced in roots at 90 min (Figure 2B). The temporal gap between the SnRK2 activation peak (30 min) and gene expression peak (90 min) likely reflects the time required for phosphorylation-dependent signaling to reach the transcriptional machinery.

In contrast to Arabidopsis, ABA responses in rice and soybean shoots were negligible under our experimental conditions. This suggests that exogenously applied ABA may not have been efficiently transported to the shoots. In Arabidopsis, ABA is synthesized in the leaf vasculature during dehydration and transported to distal tissues, and grafting experiments in tomato indicate that ABA that accumulates in roots during drought may originate from the shoots [27,28,29]. In rice, reports describing shoot responses following root-applied ABA were conducted at earlier developmental stages [30], raising the possibility that ABA transport efficiency decreases as plants mature. However, this issue remains under debate, as recent studies have also emphasized potential contributions of root-derived ABA [31]. Taken together, our results indicate that ABA translocation from roots to shoots was insufficient in rice and soybean under the conditions employed here. To enable robust interspecies comparisons of shoot responses in future work, it will be necessary to apply ABA directly to shoots, such as via foliar application or spraying, to ensure uniform hormone delivery.

Based on these results, ABA responses were clearly activated in the roots of all three species; therefore, subsequent interspecies comparisons focused on root phosphoproteomes.

### 3.2. ABA-Responsive Phosphoproteome Analysis in Arabidopsis, Rice, and Soybean

**Figure 3:**
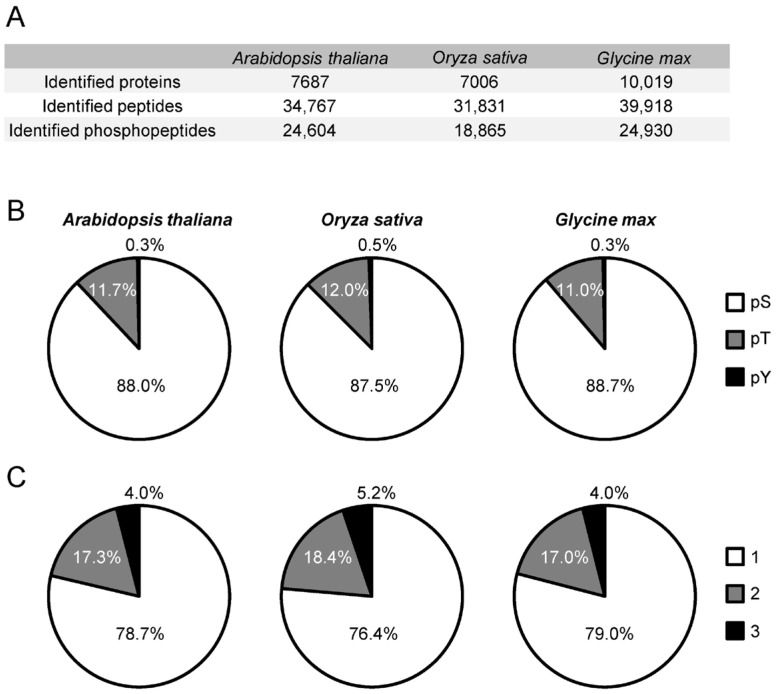
Overview of the phosphoproteomic data from Arabidopsis, rice and soybean in both shoots and roots. (**A**) Numbers of identified proteins, peptides and phosphoproteins detected by LC-MS/MS. (**B**) Distribution of phosphorylated residues in each phosphopeptides. pS, pT and pY showed phosphorylated serine, threonine and tyrosine, respectively. (**C**) Distribution of the number of phosphosites per peptide.

In this study, we conducted comparative phosphoproteome profiling of *Arabidopsis thaliana*, rice, and soybean. In total, 34,767, 31,831, and 39,918 peptides were identified from 7687 (Arabidopsis), 7006 (rice), and 10,019 (soybean) proteins, respectively (Figure 3A). Among these, 24,604, 18,865, and 24,930 were phosphorylated peptides, corresponding to HAMMOC enrichment efficiencies of 71%, 59%, and 62% (Figure 3A). The composition of phosphorylated residues (Ser/Thr/Tyr) was highly similar among the three species—88.0/11.7/0.3% in Arabidopsis, 87.5/12.0/0.5% in rice, and 88.7/11.0/0.3% in soybean (Figure 3B). The distribution of phosphorylation-site multiplicity (1/2/3 sites per peptide) was likewise comparable (78.7/17.3/4.0%, 76.4/18.4/5.2%, and 79.0/17.0/4.0%) (Figure 3C). These results demonstrate that the phosphoproteome datasets were generated with high reproducibility across species.

Recent phosphoproteomic studies of ABA signaling in Arabidopsis seedlings [32] identified 13,295 peptides (10,768 phosphopeptides), whereas our dataset contains ~2.6-fold more total peptides and ~2.3-fold more phosphopeptides, providing substantially enhanced coverage (Figure 3A). For rice, a previous ABA phosphoproteome study identified only 2162 phosphorylated peptides [17], whereas our analysis identified 18,865 (~8.7-fold increase) (Figure 3A). The higher proportion of Thr phosphorylation observed here (94/5/0.2–0.3% → 87.5/12.0/0.5%) may reflect improvements in MS sensitivity (Orbitrap Exploris 480 vs. Q-Exactive Plus) and differences in enrichment methods [17].

For soybean, a sodium bicarbonate stress phosphoproteome reported 7856 phosphorylated peptides [33], while our 24,930 peptides (~3.17-fold increase) represent substantially greater depth (Figure 3A). Although the Ser/Thr/Tyr ratios were similar, the present dataset contained a higher proportion of multiply phosphorylated peptides (previous: 1/2/3/4 = 89.4/9.6/0.9/0.1%) [33], again likely attributable to instrument sensitivity (Orbitrap Exploris 480 vs. Q-Exactive HF-X) and enrichment efficiency.

### 3.3. Analysis of the ABA-Responsive Arabidopsis Phosphoproteome

**Figure 4:**
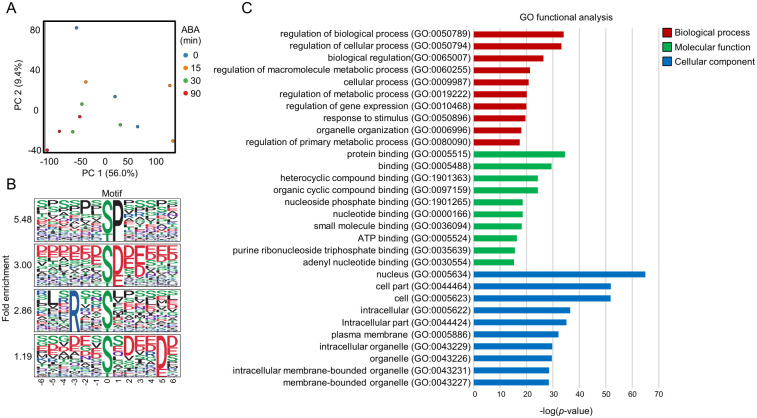
Overview of the phosphoproteomic data of Arabidopsis roots. (**A**) Principal component analysis of phosphoproteomic data from Arabidopsis roots. (**B**) Motif analysis of ABA-responsive phosphopeptides in Arabidopsis roots (fold change > 2, *p* < 0.05). Four major motif groups were identified: Group 1, [-pS/pT-P-]; Group 2, [-pS/pT-D/E-]; Group 3, [-R-x-x-pS/pT-]; and Group 4, [-pS/pT-x-x-x-x-D/E-]. (**C**) GO analysis of ABA-responsive phosphoproteins in Arabidopsis roots. GO terms were evaluated by agriGO program.

PCA of all phosphopeptides clearly separated the 0 min and 90 min samples, indicating robust phosphorylation changes at 90 min ABA treatment (Figure 4A). This observation is consistent with the in-gel kinase assay results showing maximal SnRK2 activity at 30 min (Figure 2A).

A total of 1540 phosphopeptides were significantly increased (>2-fold, *p* < 0.05) at at least one time point (15, 30, or 90 min) (Supplementary Data S1). Motif analysis using 13-amino-acid windows surrounding the phosphorylation sites identified 32 significantly enriched motifs (n > 10, *p* < 1 × 10^−6^) (Supplementary Data S6). Among these, several well-characterized motif classes were detected. Proline-directed motifs such as [-pS/pT-P-] and [-P-x-pS/pT-P-], which are typical substrates of MAPKs and certain RLKs, were prominently enriched (Figure 4B). Acidic motifs including [-pS/pT-D/E-], [-pS/pT-x-D/E-], and [-pS/pT-D/E-x-D/E-]—commonly associated with CK2-type kinases [34]—were also abundant (Figure 4B). In addition, basic SnRK2-type motifs such as [-R-x-x-pS/pT-] and [-L-x-R-x-x-pS/pT-] were enriched, along with acidic-tail–type motifs (e.g., [-pS/pT-x-x-x-x-D/E-]) (Figure 4B), consistent with previously reported consensus sequences for SnRK2 substrates. The proline-directed motif was the most frequently identified, consistent with its general abundance in plant phosphoproteomes [11,35]. The enrichment of both SnRK2-type and CK2-type acidic motifs suggests the coordinated activation of multiple kinase families during ABA signaling.

GO enrichment of proteins harboring ABA-increased phosphopeptides revealed significant overrepresentation of biological processes related to metabolism, gene expression, and intracellular signaling (Figure 4C). In Cellular Component, the nucleus was the most enriched category, indicating that a substantial proportion of ABA-responsive phosphorylation events occur in nuclear proteins. This is consistent with established models in which SnRK2s phosphorylate transcription factors and nuclear regulatory proteins to initiate ABA-responsive gene expression.

### 3.4. Overview of the ABA-Responsive Phosphoproteome in Rice

**Figure 5:**
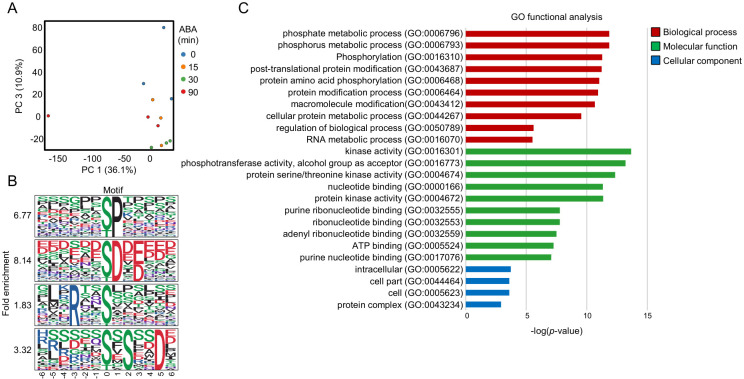
Overview of the phosphoproteomicdata of rice roots. (**A**) Principal component analysis of phosphoproteomic data from rice roots. (**B**) Motif analysis of ABA-responsive phosphopeptides in Arabidopsis roots (fold change > 2, *p* < 0.05). Four major motif groups were identified: Group 1, [-pS/pT-P-]; Group 2, [-pS/pT-D-]; Group 3, [-R-x-x-pS/pT-]; and Group 4, [-pS/pT-x-S-x-x-D-]. (**C**) GO analysis of ABA-responsive phosphoproteins in rice roots. GO terms were evaluated by agriGO program.

PCA revealed that samples treated with ABA for 30 min were clearly separated from the 0 min samples, indicating that substantial ABA-dependent phosphorylation changes occurred at this time point (Figure 5A). A total of 1317 phosphopeptides were significantly increased upon ABA treatment, and motif analysis identified 18 significantly enriched motifs (Supplementary Data S6). Overall, these motifs resembled those identified in Arabidopsis (Figure 5B).

Notably, the [-pS-F-] motif—previously reported as a characteristic motif in rice leaves, seeds, and seedlings [17,36,37]—was not detected in our dataset. This absence may reflect differences in tissue type (roots vs. aboveground tissues) or indicate that ABA-dependent phosphorylation in rice roots uses a distinct set of substrates compared with previous studies.

GO enrichment analysis showed that biological process terms related to phosphorylation regulation were significantly enriched, and kinase activity was among the top-ranked Molecular function terms (Figure 5C). These results suggest that multiple kinase pathways are activated during the rice ABA response.

### 3.5. Overview of the ABA-Responsive Phosphoproteome in Soybean

**Figure 6:**
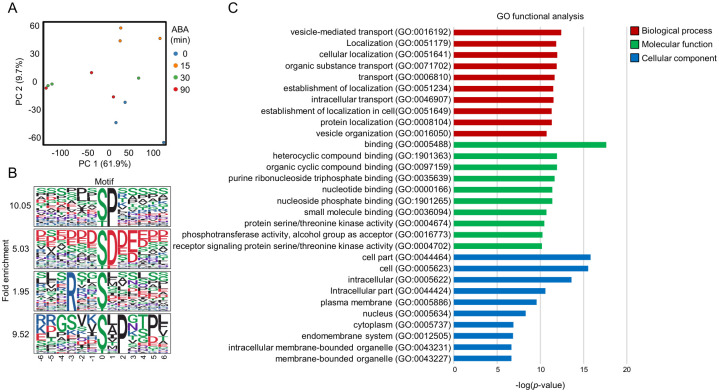
Overview of the phosphoproteomicdata of soybean root. (**A**) Principal component analysis of phosphoproteomic data from soybean roots. (**B**) Motif analysis of ABA-responsive phosphopeptides in soybean roots (fold change > 2, *p* < 0.05). Four major motif groups were identified: Group 1, [-pS/pT-P-]; Group 2, [-pS/pT-D/E-]; Group 3, [-R-x-x-pS/pT-]; and Group 4, [-pS/pT-x-P-]. (**C**) GO analysis of ABA-responsive phosphoproteins in soybean roots. GO terms were evaluated by agriGO program.

PCA showed that the 15 min ABA-treated samples were already separated from the 0 min samples (Figure 6A), suggesting that phosphorylation changes occurred earlier in soybean than in the other species. However, SnRK2 activation kinetics measured by in-gel kinase assay were similar to those of Arabidopsis, indicating that the early PCA separation may reflect species-specific characteristics or differences in the onset of substrate phosphorylation.

ABA treatment significantly increased 2175 phosphopeptides, and motif analysis identified 41 significant motifs (n > 10, *p* < 1 × 10^−6^) (Supplementary Data S6). While the majority of motifs overlapped with those found in Arabidopsis and rice, a notable difference was that the acidic tail motif [-pS/pT-x-x-x-x-D/E-], which was strongly enriched in Arabidopsis, was not enriched in soybean (Figure 6B). Conversely, nine motifs previously reported as legume-specific (e.g., [-pS-P-K-], [-pS-P-R-]; [38]) were reproducibly detected (Supplementary Data S6), supporting the idea that certain phosphorylation features are conserved across legumes.

GO analysis in soybean indicated enrichment of biological process terms related to vesicle-mediated transport and organic substance transport (Figure 6C). However, no clear pattern of GO enrichment was shared among all three species. This lack of shared GO signatures likely reflects limitations in cross-species annotation, as functional annotation in soybean and rice remains less comprehensive than in Arabidopsis.

### 3.6. Inter-Species Comparison of ABA-Induced Phosphorylation Dynamics

**Figure 7:**
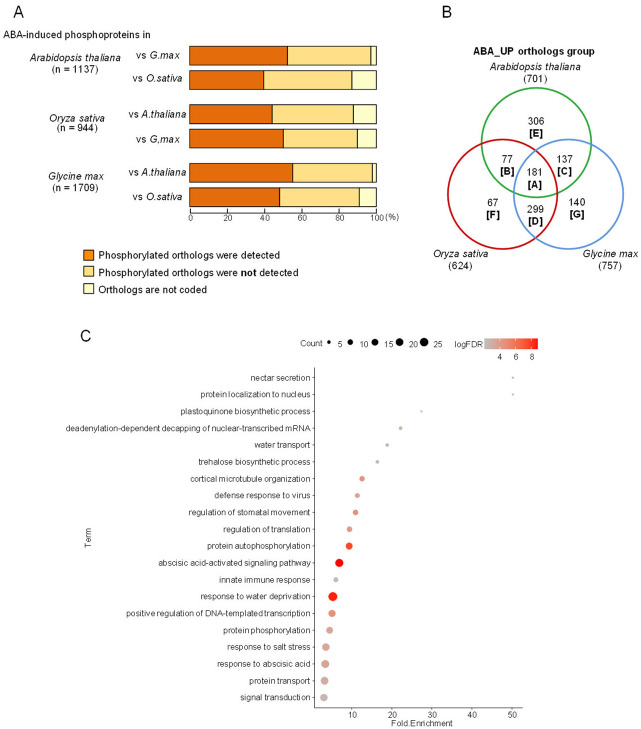
Interspecies comparative analysis of ABA-induced phosphoproteins. (**A**) Orthologous analysis of ABA-induced phosphoproteins among Arabidopsis, rice, and soybean. Orthologous protein groups were identified using the OrthoMCL algorithm. ABA-induced phosphoproteins whose orthologs also showed ABA-induced phosphorylation in other species are indicated in red boxes. ABA-induced phosphoproteins whose orthologs did not show ABA-induced phosphorylation are shown in pink boxes, and proteins without identifiable orthologs are shown in gray boxes. (**B**) Venn diagram showing overlap of ortholog groups with ABA-induced phosphorylation among Arabidopsis, rice, and soybean. (**C**) GO analysis of ABA-responsive phosphoproteins conserved across all three species. Arabidopsis genes belonging to Group A ortholog groups in (**B**) were used for GO analysis. Biological process terms were evaluated using the DAVID program.

To compare ABA-responsive phosphorylation across the three species, ortholog groups for Arabidopsis and rice were retrieved from OrthoMCL [20], and soybean genes were subsequently assigned to these groups using the same algorithm, as soybean is not included in the original OrthoMCL dataset. ABA treatment increased phosphorylation in 1137 Arabidopsis proteins, 944 rice proteins, and 1709 soybean proteins (Figure 7A). Among the 1137 Arabidopsis ABA-responsive proteins, 97% (1103) had orthologs in soybean and 86.9% (988) had orthologs in rice, and ABA-induced phosphorylation was observed in 52.3% of the soybean orthologs (595 proteins) and 39.7% of the rice orthologs (451 proteins) (Figure 7A). These values indicate that conservation of ABA-responsive phosphorylation is higher between the two dicots (Arabidopsis and soybean) than between Arabidopsis and the monocot rice. A previous global phosphoproteomics study [14] reported that 56.2% of Arabidopsis phosphoproteins had phosphorylated orthologs in rice; therefore, the lower conservation observed here for ABA-responsive phosphoproteins suggests that hormone-responsive phosphorylation is less conserved than global phosphorylation dynamics. When rice was used as the reference, ABA-responsive phosphorylation was conserved in 44.2% of Arabidopsis orthologs (417 proteins) and in 50.1% of soybean orthologs (473 proteins) (Figure 7A). Soybean consistently displayed the highest number of ABA-responsive phosphoproteins, suggesting that ABA signaling in this species may involve a broader phosphorylation network.

After assigning all ABA-responsive proteins to ortholog groups, interspecies overlap was visualized using a Venn diagram (Figure 7B). The number of ABA-responsive ortholog groups was 701 in Arabidopsis, 624 in rice, and 757 in soybean (Figure 7B, Supplementary Data S5). The greatest overlap was observed between rice and soybean: 76.9% (480) of the rice ABA-responsive ortholog groups also exhibited ABA-induced phosphorylation in soybean (Figure 7B). Species-specific ABA-responsive ortholog groups were most abundant in Arabidopsis (306 groups), while rice and soybean had 67 and 140 species-specific groups, respectively (Figure 7B). A total of 181 ortholog groups showed ABA responsiveness in all three species, representing approximately 24–29% of responsive groups in each species (Figure 7B).

Gene ontology analysis of these universally conserved ortholog groups (Group A) revealed strong enrichment for biological processes associated with ABA signaling and drought responses, such as “abscisic acid-activated signaling pathway” and “response to water deprivation,” indicating that conserved ABA-responsive phosphorylation events correspond to canonical stress-response pathways (Figure 7C). Enrichment in protein kinase activity and serine/threonine kinase activity further highlights the central role of multilayered kinase cascades in ABA signaling across land plants (Figure 7C). Cellular component terms, including nucleus, cytoplasm, and plasma membrane, were also significantly enriched (Figure 7C), consistent with a model wherein ABA signaling relies on coordinated phosphorylation events occurring across multiple intracellular compartments.

OrthoMCL proved effective for systematic comparisons of evolutionary conservation and species specificity among ABA-responsive phosphoproteins, and it enabled functional inferences for uncharacterized genes based on their orthologous relationships, consistent with earlier applications of OrthoMCL in studies such as the identification of soybean flowering regulators [39]. Nonetheless, because OrthoMCL groups paralogous genes into the same ortholog clusters, the overlap illustrated in Figure 7B reflects ortholog groups rather than individual genes. Thus, OrthoMCL is more suitable for qualitative assessment of evolutionary patterns than for quantitative comparisons of gene numbers. Future work will focus on identifying genes with strictly conserved ABA-responsive phosphorylation sites across species, characterizing those with divergent or species-specific sites, and elucidating the molecular evolutionary features that have shaped ABA-responsive phosphorylation networks. Such analyses will deepen our understanding of how ABA signaling pathways have diversified across monocots and dicots while preserving their conserved regulatory cores.

### 3.7. Conservation of Phosphorylation Sites in ABA Signaling Factors

**Figure 8:**
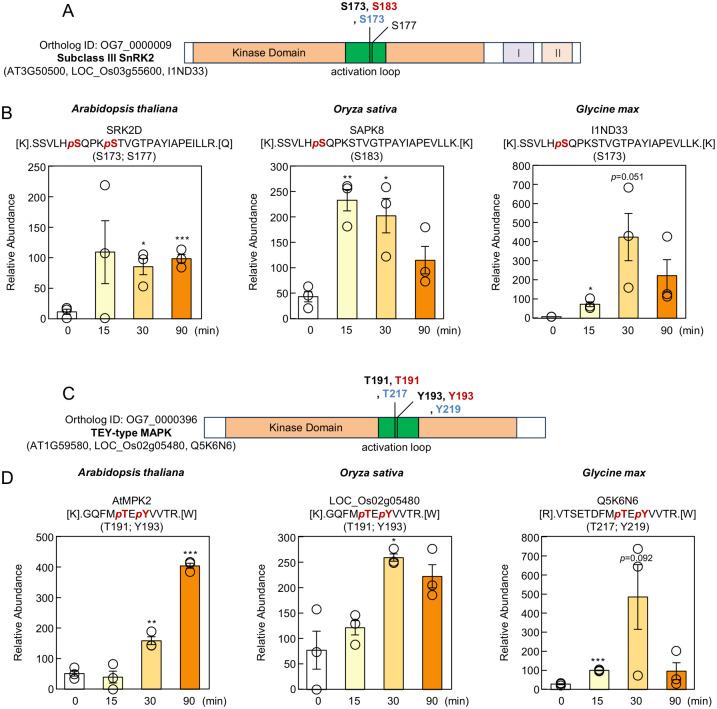
ABA-induced phosphorylation sites of core signaling factors are widely conserved across species. (**A**) Domain structures of SnRK2 proteins. ABA-induced phosphorylation sites identified in this study are indicated by black (Arabidopsis), red (rice), or blue (soybean) lines (fold change > 2, *p* < 0.05). (**B**) Relative abundance of phosphopeptides containing phosphorylated Ser180 and Ser184 in SRK2D (Arabidopsis), Ser183 in SAPK8 (rice), and Ser173 in I1ND33 (soybean). Data represent means ± SE (*n* = 3 biologically independent samples). Asterisks indicate statistically significant differences as determined by Student’s *t*-test (* *p* < 0.05, ** *p* < 0.01, *** *p* < 0.001; not significant, *p* > 0.05). (**C**) Domain structures of MAP kinases. ABA-induced phosphorylation sites identified in this study are indicated by black (Arabidopsis), red (rice), or blue (soybean) lines (fold change > 2, *p* < 0.05). (**D**) Relative abundance of phosphopeptides containing phosphorylated Thr191 and Tyr193 in AtMPK2 (Arabidopsis), Thr191 and Tyr193 in LOC_Os02g05480 (rice), and Thr217 and Tyr219 in Q5K6N6 (soybean). Data are presented as means ± SE (*n* = 3 biologically independent samples). Statistical significance was determined by Student’s *t*-test (* *p* < 0.05, ** *p* < 0.01, *** *p* < 0.001; not significant, *p* > 0.05).

We first examined the interspecies conservation of ABA-responsive phosphorylation sites in group A proteins. Ortholog ID OG7_0000009 contains subclass III SnRK2 kinases. In Arabidopsis, SRK2D/SnRK2.2 exhibited a pronounced increase in phosphorylation of a peptide containing S173 and S177 within the activation loop at 30 and 90 min after ABA treatment (Figure 8A,B), consistent with previous reports [11]. In SAPK8 (rice) and I1ND33 (soybean), which belong to the same ortholog group, phosphorylation at S183 (SAPK8) and S173 (I1ND33) increased from 15 min after ABA application (Figure 8A,B). These were the only ABA-induced SnRK2 phosphorylation sites identified. Although S7, S30, and S153 were also phosphorylated in Arabidopsis SRK2D, no ABA-dependent increase was detected. The site corresponding to SRK2D/SnRK2.2 S173 is essential for SnRK2 activation by Raf-like kinases [40,41]. To assess evolutionary conservation, full-length SnRK2 sequences from ten plant species—including algae (Chlamydomonas), mosses, ferns, and angiosperms—were aligned (Supplementary Figure S12). Phosphorylation sites in the activation loop were highly conserved across land plants (Supplementary Figure S12). In our phosphoproteomics, SnRK2 phosphorylation increased at 15 min and remained elevated at 90 min after ABA treatment, whereas in-gel kinase activity decreased at 90 min (Figure 2A and Figure 8B). Because mass spectrometry quantifies phosphorylation levels while kinase assays measure catalytic activity toward substrates, these different metrics may explain the observed discrepancy.

We next examined OG7_0000396, which contains mitogen-activated protein kinases (MAPKs). Arabidopsis AtMPK1/2 are known to be phosphorylated at the conserved TEY motif in response to ABA and dehydration [11], and subclass III SnRK2s are required for their activation. In this study, phosphorylation at T191 and Y193 (the TEY motif in AtMPK2) increased at 30 and 90 min after ABA treatment (Figure 8C,D). Likewise, phosphorylation of TEY-containing peptides in rice LOC_Os02g05480 and soybean Q5K6N6 increased from 15 min (Figure 8C,D). Multiple sequence alignment confirmed that the kinase domain, including TEY motif, is highly conserved across land plants (Supplementary Figure S13), consistent with previous reports [42].

**Figure 9:**
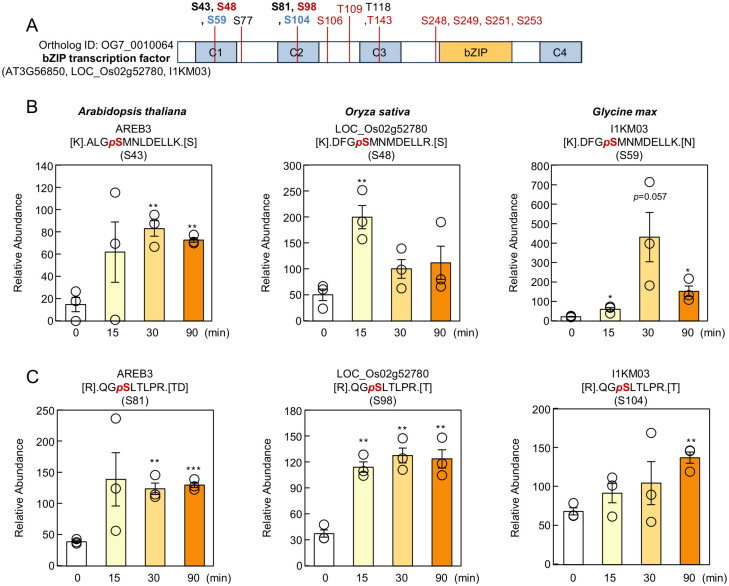
Phosphorylation sites of bZIP transcription factors are partially conserved across species. (**A**) Domain structure of bZIP transcription factors. ABA-induced phosphorylation sites identified in this study are indicated by black (Arabidopsis), red (rice), or blue (soybean) lines (fold change > 2, *p* < 0.05). (**B**) Relative abundance of phosphopeptides containing phosphorylated Ser43 in AREB3 (Arabidopsis), Ser48 in LOC_Os02g52780 (rice), and Ser59 in I1KM03 (soybean). (**C**) Relative abundance of phosphopeptides containing phosphorylated Ser81 in AREB3 (Arabidopsis), Ser98 in LOC_Os02g52780 (rice), and Ser104 in I1KM03 (soybean). Data are presented as means ± SE (*n* = 3 biologically independent samples). Asterisks indicate statistically significant differences (* *p* < 0.05, ** *p* < 0.01, *** *p* < 0.001; not significant, *p* > 0.05).

We also analyzed OG7_0010064, which contains bZIP transcription factors of the AREB/ABF family. AREB proteins bind ABREs to activate ABA-responsive gene expression [2] and harbor four conserved domains (C1–C4) containing multiple [-R-x-x-S-] motifs that are targeted by SnRK2s [9]. In this study, phosphorylation of AREB3 S43 increased at 30 and 90 min after ABA treatment (Figure 9A,B), in agreement with earlier findings showing SnRK2-dependent phosphorylation of AREB1 S45 [11]. In rice and soybean, phosphorylation at S48 and S59, respectively—sites corresponding to AREB3 S43—also increased from 15 min. Phosphorylation at AREB3 S81 (corresponding to rice S98 and soybean S104) similarly increased in response to ABA (Figure 9A,C).

Although additional ABA-induced phosphorylation sites were detected in Arabidopsis AREB3 and rice LOC_Os02g52780 (Figure 9A), none were shared across all three species. Alignment of AREB homologs from ten plant species revealed strong conservation of the C1–C4 domains (Supplementary Figure S14). Importantly, S43 was conserved in all examined species, while S81 was conserved in all except Chlamydomonas. These findings, together with the phosphorylation dynamics observed here, suggest that S43 and S81 represent core regulatory sites essential for AREB function.

### 3.8. ABA-Responsive Phosphorylation Events Lacking Interspecies Conservation

**Figure 10:**
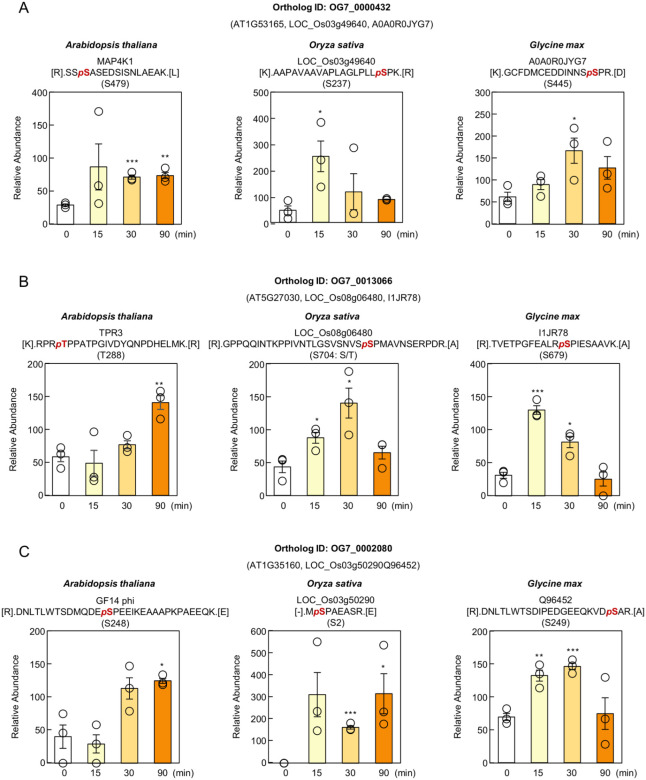
Species-specific ABA-induced phosphorylation sites within individual ortholog groups. (**A**–**C**) Relative abundance of phosphopeptides containing phosphorylated Ser479 in MAP4K1 (Arabidopsis), Ser237 in LOC_Os03g49640 (rice), and Ser445 in A0A0R0JYG7 (soybean) (**A**); phosphorylated Thr288 in TPR3 (Arabidopsis), Ser704 in LOC_Os08g06480 (rice), and Ser679 in I1JR78 (soybean) (**B**); and phosphorylated Ser248 in GRF4 (Arabidopsis), Ser2 in LOC_Os03g50290 (rice), and Ser249 in Q96452 (soybean) (**C**). Data are presented as means ± SE (*n* = 3 biologically independent samples). Statistical significance was determined by Student’s *t*-test (* *p* < 0.05, ** *p* < 0.01, *** *p* < 0.001; not significant, *p* > 0.05).

We also examined Group A orthologs that did not share ABA-responsive phosphorylation sites across species. OG7_0000432 contains MAP4K kinases, and in Arabidopsis, MAP4K1 S479 is directly phosphorylated by subclass III SnRK2s and promotes ABA-induced stomatal closure through Ca^2+^ channel activation [43]. In our dataset, phosphorylation at S479 increased upon ABA treatment in Arabidopsis (Figure 10A). However, the ABA-responsive sites detected in rice (S237) and soybean (S445) did not correspond to S479, suggesting that MAP4K phosphorylation is controlled differently among species. A similar pattern was observed for TPR3 (Ortholog ID OG7_0013066), a member of the TOPLESS/TOPLESS-related corepressor family [44,45]. Arabidopsis exhibited ABA-induced phosphorylation at T288 (Figure 10B), while rice and soybean showed phosphorylation at S704 and S679, respectively, and neither residue was conserved with the Arabidopsis site. Species-specific regulation was also evident in GF14 phi (Ortholog ID OG7_0002080), a 14-3-3 protein involved in diverse hormone and stress responses [46,47]. In Arabidopsis, S248 was phosphorylated in response to ABA (Figure 10C), whereas in rice and soybean, phosphorylation occurred at S2 and S249, respectively, and neither site corresponded to the Arabidopsis residue. These findings indicate that even when orthologous proteins are conserved across species, the specific residues targeted by ABA-responsive kinases can diverge substantially.

To characterize the broader features of Groups B–G, we extracted Arabidopsis genes belonging to each ortholog group and performed GO enrichment analysis (Supplementary Figures S2, S4 and S6–S9). Group B, shared between Arabidopsis and rice, was enriched for auxin polar transport and auxin-activated signaling pathways, with representative phosphorylation patterns shown for OG7_0009290, OG7_0003946, and OG7_0006260 in Supplementary Figures S2 and S3. Group C, shared between Arabidopsis and soybean, showed enrichment for negative regulation of DNA-templated transcription and defense responses to bacteria, and examples of phosphorylation profiles for OG7_0010633, OG7_0001365, and OG7_0000402 are provided in Supplementary Figures S4 and S5. Group D, shared between rice and soybean, was enriched for regulation of DNA-templated transcription and responses to salt stress, with representative cases illustrated by OG7_0011124, OG7_0022264, and OG7_0001733 (Supplementary Figures S6 and S7). Because functional genomic resources for rice and soybean remain less extensive than those for Arabidopsis, identifying common biological themes within Groups B–D remains challenging.

Species-specific ABA-responsive phosphoproteins were classified into Groups E–G (Figure 7B). Group E, consisting of Arabidopsis-specific genes, was enriched for functions associated with the reductive pentose-phosphate cycle and circadian rhythm (Supplementary Figure S8). Group F, which is rice-specific, showed enrichment for mRNA catabolic processes and ABA responses, while Group G, specific to soybean, was enriched for protein lipoylation and vesicle-mediated transport (Supplementary Figures S9 and S10). Quantitative information on phosphorylation sites and ABA responsiveness for Groups E–G is summarized in Supplementary Figure S11.

## 4. Conclusions

The entire proteome encompasses not only mature proteins but also a wide range of proteoforms, including those produced by processing, post-translational modifications (such as phosphorylation and glycosylation), and various protein variants within cells and tissues. Given that multiple protein forms can arise from a single gene and change dynamically across time and space, the proteome is inherently extraordinarily complex.

This study provides a comprehensive overview of the ABA-responsive phosphorylation landscape across Arabidopsis, rice, and soybean. Although ABA-responsive phosphoproteome profiles in soybean have been limited, our analysis demonstrated that the core ABA signaling components—such as SnRK2 kinases and bZIP transcription factors—are well conserved across all three species (Figure 8 and Figure 9). We also identified 181 ortholog groups that are commonly phosphorylated in response to ABA in three species (Figure 7B). Furthers studies will be required to determine which of these phosphoproteins function in ABA responses in plants. Among the proteins whose phosphorylation levels increased in all three species following ABA treatment, many exhibited species-specific phosphorylation sites. (Figure 10), indicating that ABA-responsive regulatory networks have diversified during plant evolution.

In this study, we used label-free quantification (LFQ) LC-MS/MS phosphoproteomics. This method is common for investigating protein phosphorylation, but it has some limitations. One major problem is the random sampling in data-dependent acquisition (DDA). This often causes missing values and incomplete datasets. LFQ is also very sensitive to changes in the instrument and sample preparation. These issues can lower accuracy and reproducibility. In addition, low abundance phosphopeptides are hard to detect, so the coverage of the phosphoproteome is not complete.

It should also be noted that we focused on the ABA responses at relatively early developmental stages. ABA plays organ-specific roles across diverse stages of plant growth, including seed dormancy, stomatal closure, flowering, and senescence. Our phosphoproteomic data reveal only a part of this complexity, and elucidating the stage- and tissue-specific regulation of phosphorylation mediated by ABA remains an important subject for future investigation.

A future challenge is to quantitatively compare ABA-responsive phosphorylation pathways with those activated by osmotic stress. Integration of phosphorylation proteome datasets across stress conditions will help establish a more complete model of plant stress signaling. Moreover, extending this comparative framework to additional plant species and environmental stimuli may identify phosphorylation motifs that are deeply conserved across the plant kingdom, as well as lineage-specific regulatory innovations.

## Data Availability

LC-MS/MS raw data of phosphoproteomic analysis have been deposited in Japan Proteome Standard Repository/Database (jPOSTP) [48]. Phosphoproteomics of Arabidopsis; link (https://repository.jpostdb.org/preview/152489080269364d5ad0e19, accessed on 10 December 2025), Access key (4041), rice; link (https://repository.jpostdb.org/preview/1378178452693b4e8be491c, accessed on 10 December 2025), Access key (4478), soybean; link (https://repository.jpostdb.org/preview/307350493693b4eb503a4e, accessed on 10 December 2025), Access key (8035).

## Supplementary Materials

The following supporting information can be downloaded at: https://www.mdpi.com/article/10.3390/proteomes14010004/s1, Figure S1: GO analysis of ABA-responsive phosphoproteins in Arabidopsis, rice and soybean root; Figure S2: GO analysis of ABA-responsive phosphoproteins in Arabidopsis root and rice root; Figure S3: ABA-responsive phosphoproteins in both Arabidopsis and rice; Figure S4: GO analysis of ABA-responsive phosphoproteins in Arabidopsis root and soybean root; Figure S5: ABA-responsive phosphoproteins in both Arabidopsis and soybean; Figure S6: GO analysis of ABA-responsive phosphoproteins in rice root and soybean root; Figure S7: ABA-responsive phosphoproteins in both rice and soybean; Figure S8: GO analysis of ABA-responsive phosphoproteins in Arabidopsis root; Figure S9: GO analysis of ABA-responsive phosphoproteins in rice root; Figure S10: GO analysis of ABA-responsive phosphoproteins in soybean root; Figure S11: ABA-responsive phosphoproteins only in Arabidopsis, rice or soybean; Figure S12: Alignment of full length SnRK2s in species of Arabidopsis, rice, soybean, Solanum lycopersicum, Populus, Zea mays, Selaginella moellendorffii, Physcomitrella patens, Marchantia polymorpha, Chara braunii; Figure S13: Alignment of full length MAPK in species of Arabidopsis, rice, soybean, Solanum lycopersicum, Populus, Zea mays, Selaginella moellendorffii, Physcomitrella patens, Marchantia polymorpha, Chara braunii; Figure S14: Alignment of full length bZIP transcription factor in species of Arabidopsis, rice, soybean, Solanum lycopersicum, Populus, Zea mays, Selaginella moellendorffii, Physcomitrella patens, Marchantia polymorpha, Chara braunii; Data S1: Phosphopeptide abundances in Arabidopsis; Data S2: Phosphopeptide abundances in rice; Data S3: Phosphopeptide abundances in soybean; Data S4: Classification of Arabidopsis, rice and soybean genes into orthologous groups; Data S5: Orthologous analysis of ABA-induced phosphoproteins among Arabidopsis, rice, and soybean; Data S6: Motif analysis of phosphopeptides increased by ABA treatment in Arabidopsis, rice and soybean; Data S7: Primer sequences used for RT-qPCR.
