# Supplementary material for: Cross-Species Analysis of ABA-Induced Phosphosignaling Landscapes in Rice, Soybean, and Arabidopsis
Source: Proteomes. 2026 Jan 20;14(1):4. doi: 10.3390/proteomes14010004 (PMC12922155; doi:10.3390/proteomes14010004)
Supplement: Supplementary file 1 [file proteomes-14-00004-s001.zip › 260105_Proteome_Figure_Sup.pdf]

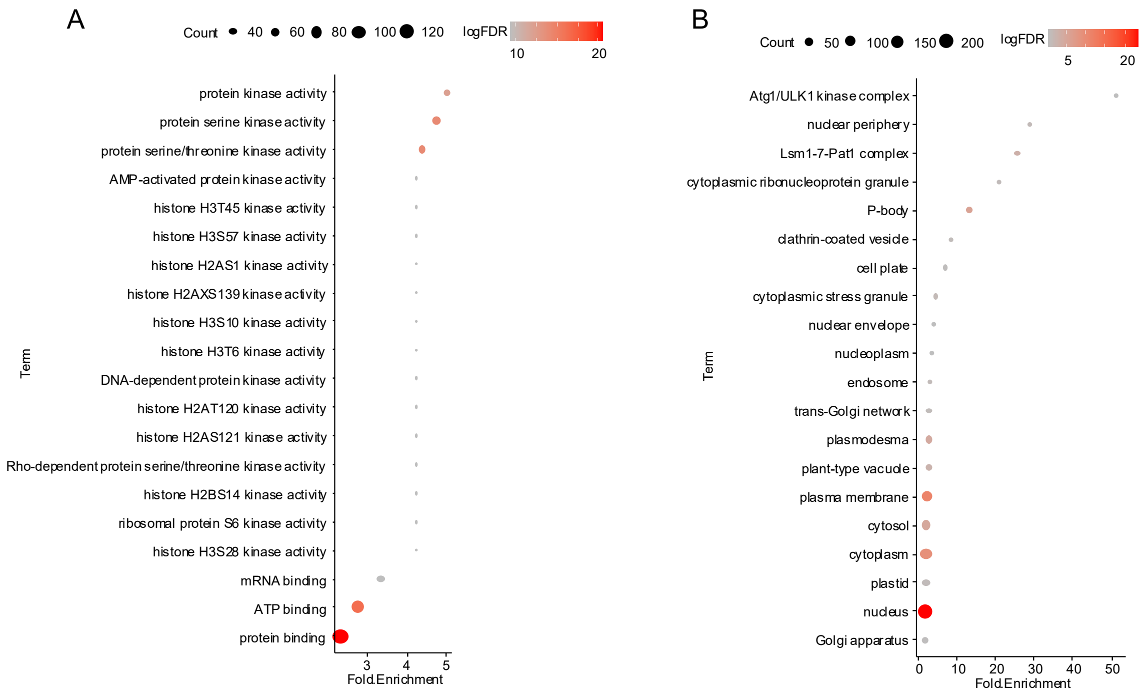

Figure.S1 GO analysis of ABA-responsive phosphoproteins in Arabidopsis, rice and soybean root.

Arabidopsis genes included group A orthologous groups at Figure.6B were used for GO analysis. GO terms of molecular function(A) and cellular component(B)were evaluated by DAVID program.

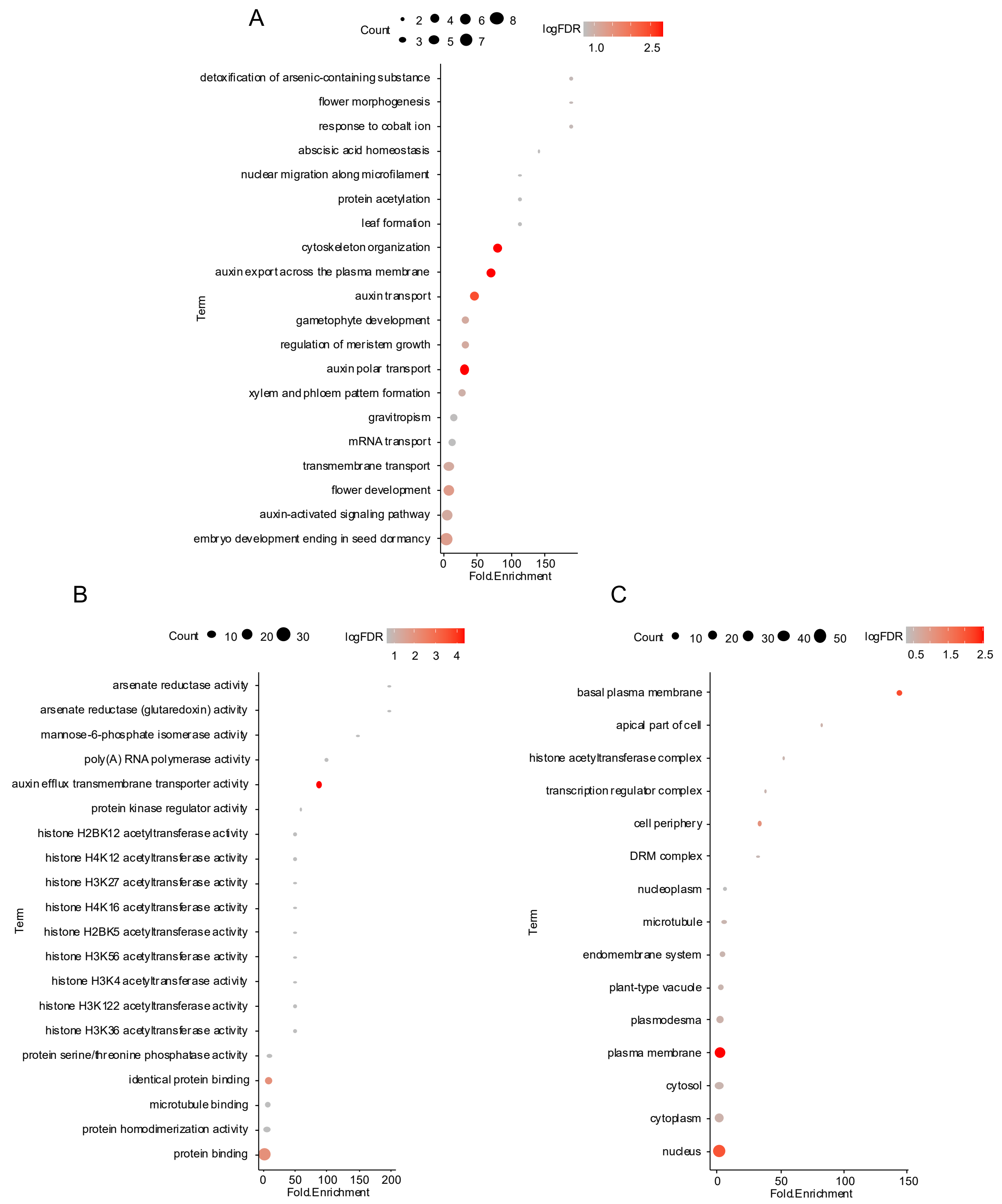

Figure.S2 GO analysis of ABA-responsive phosphoproteins in Arabidopsis root and rice root. Arabidopsis genes included group B orthologous groups at Figure.6B were used for GO analysis. GO terms of biological process(A), molecular function(B) and cellular component(C)were evaluated by DAVID program.

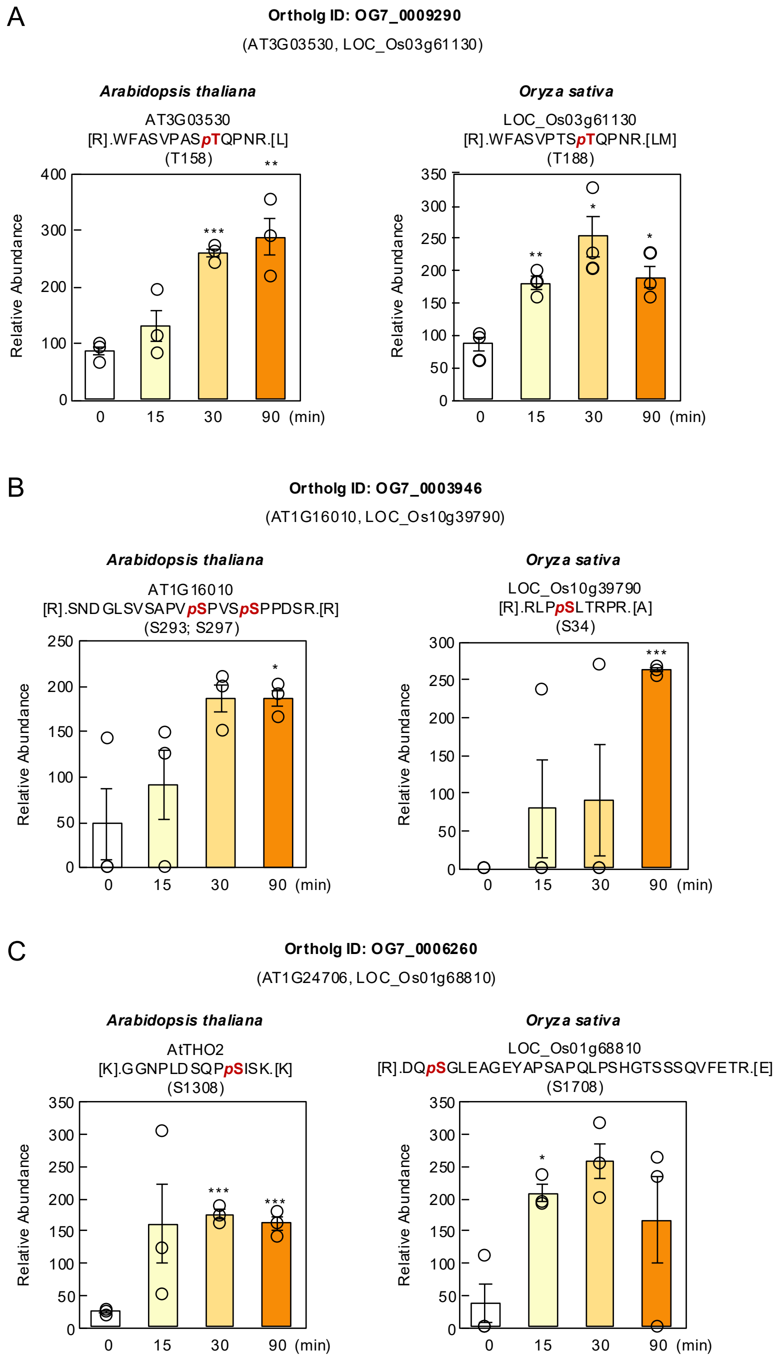

Figure.S3 ABA-responsive phosphoproteins in both Arabidopsis and rice.
(A, B and C)Relative abundance of phosphopeptides containing phosphorylated Thr158 in AT3G03530 (Arabidopsis), Thr188 in LOC_Os03g61130 (rice) (A). Relative abundance of phosphopeptides containing phosphorylated Ser293 and Ser297 in AT1G16010 (Arabidopsis), Ser34 in LOC_Os10g39790 (rice)(B). Relative abundance of phosphopeptides containing phosphorylated Ser1308 in AtTHO2 (Arabidopsis), Ser1708 in LOC_Os01g68810 (rice)(C). Data are presented as mean ± SE (n = 3 biologically independent samples), and asterisks indicate significant differences as determined by Student's t-test (**P* < 0.05, ***P* < 0.01; not significant *P* > 0.05).

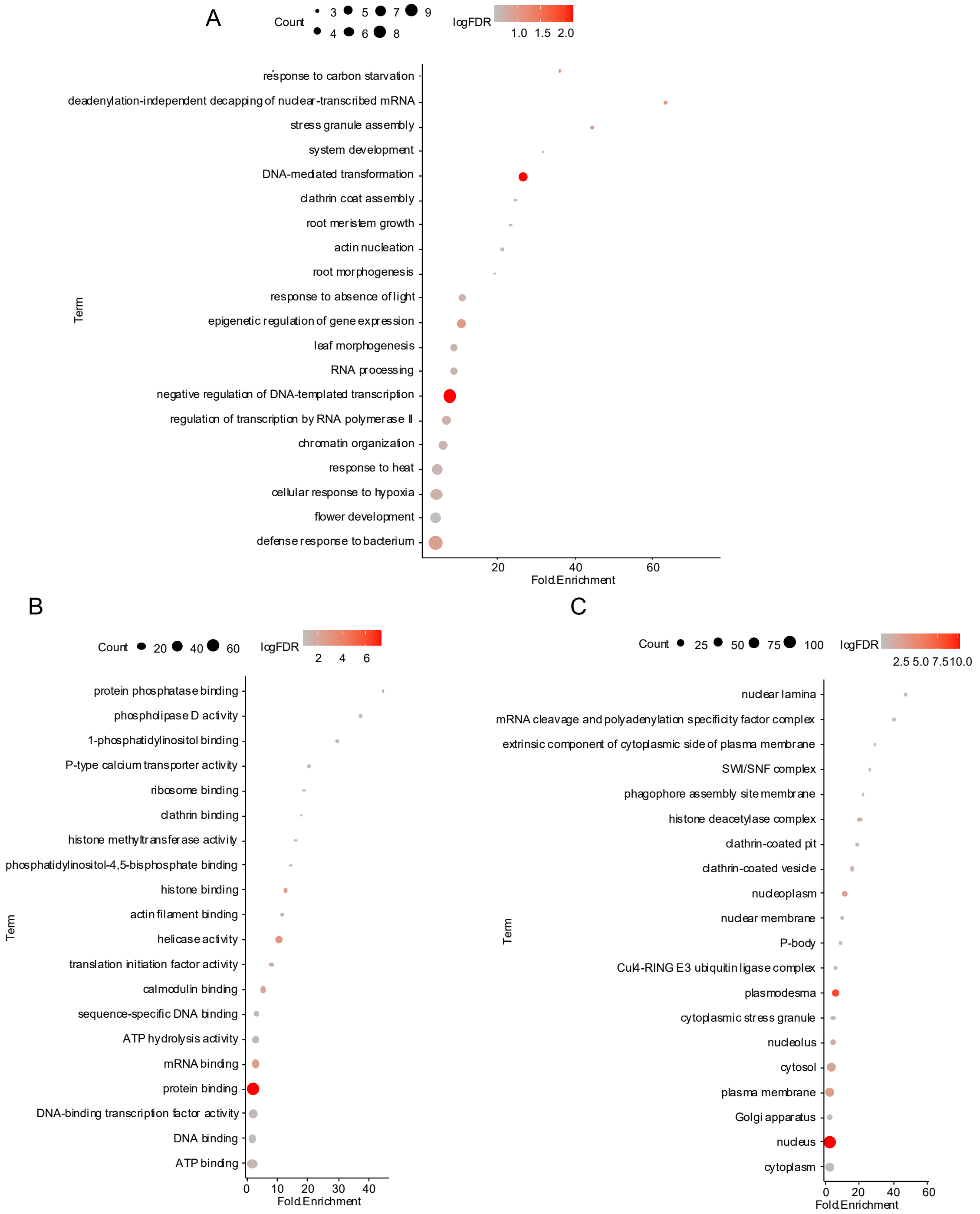

Figure.S4 GO analysis of ABA-responsive phosphoproteins in Arabidopsis root and soybean root. Arabidopsis genes included group C orthologous groups at Figure.6B were used for GO analysis. GO terms of biological process(A), molecular function(B) and cellular component(C)were evaluated by DAVID program.

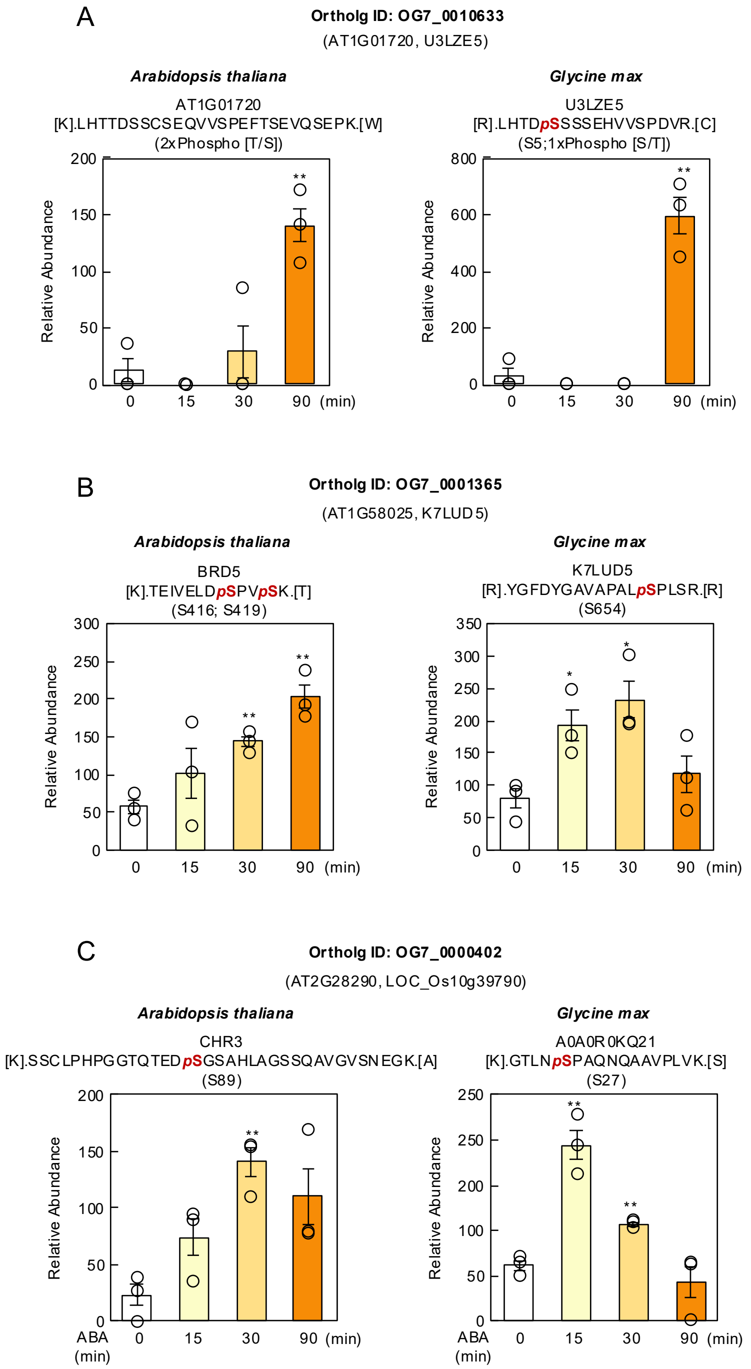

Figure.S5 ABA-responsive phosphoproteins in both Arabidopsis and soybean.
(A, B and C)Relative abundance of phosphopeptides in AT1G01720 (Arabidopsis), Ser5 in U3LZE5 (soybean) (A). Relative abundance of phosphopeptides containing phosphorylated Ser416 and Ser419 in BRD5 (Arabidopsis), Ser654 in K7LUD5 (soybean)(B). Relative abundance of phosphopeptides containing phosphorylated Ser89 in CHR3 (Arabidopsis), Ser27 in A0A0R0KQ21 (soybean)(C). Data are presented as mean ± SE (n = 3 biologically independent samples), and asterisks indicate significant differences as determined by Student's t-test (**P* < 0.05, ***P* < 0.01; not significant *P* > 0.05).

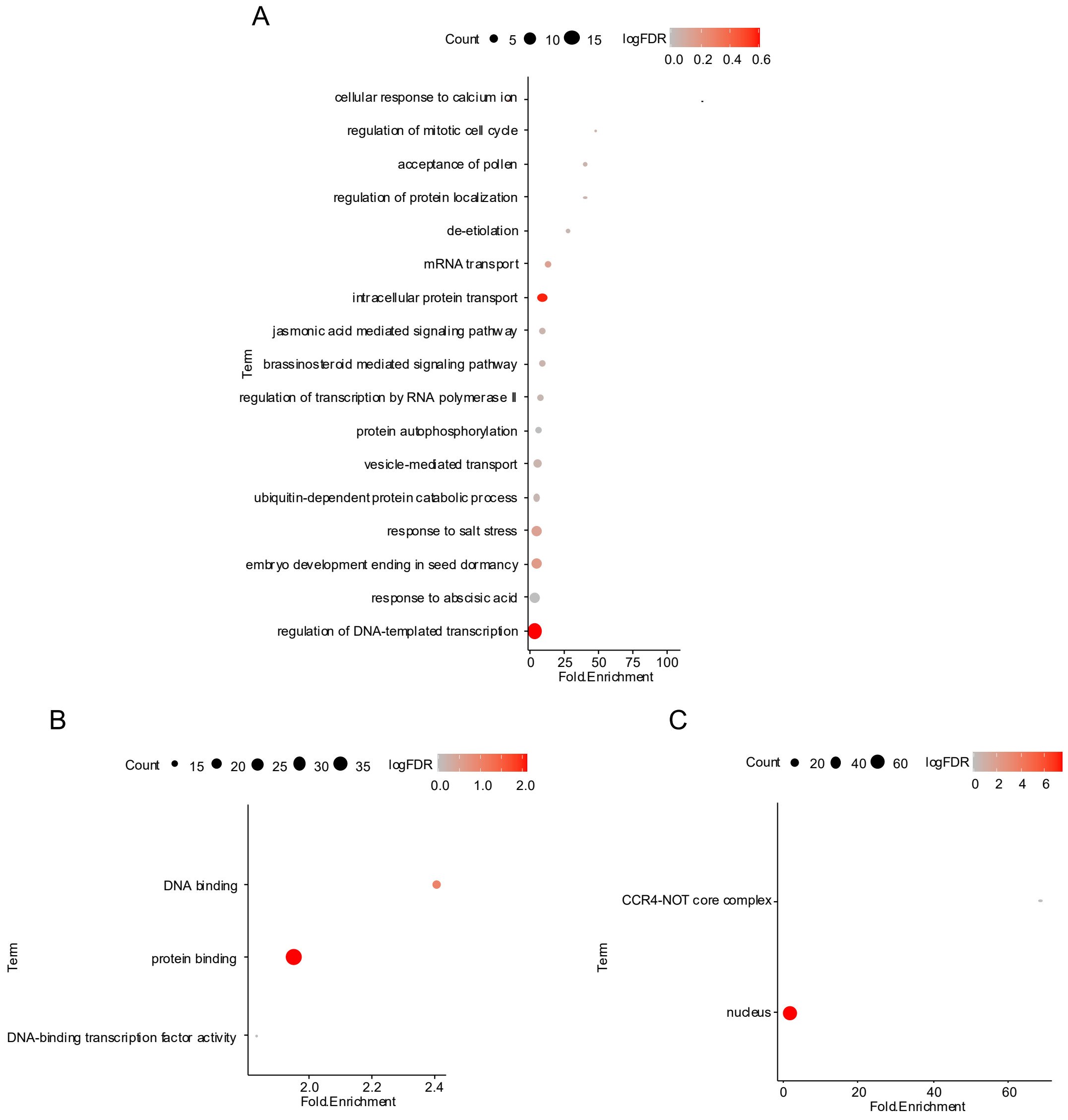

Figure.S6 GO analysis of ABA-responsive phosphoproteins in rice root and soybean root.
Genes included group D orthologous groups at Figure 6B were translated representative Arabidopsis genes and used for GO analysis. GO terms of biological process(A), molecular function(B) and cellular component(C)were evaluated by DAVID program.

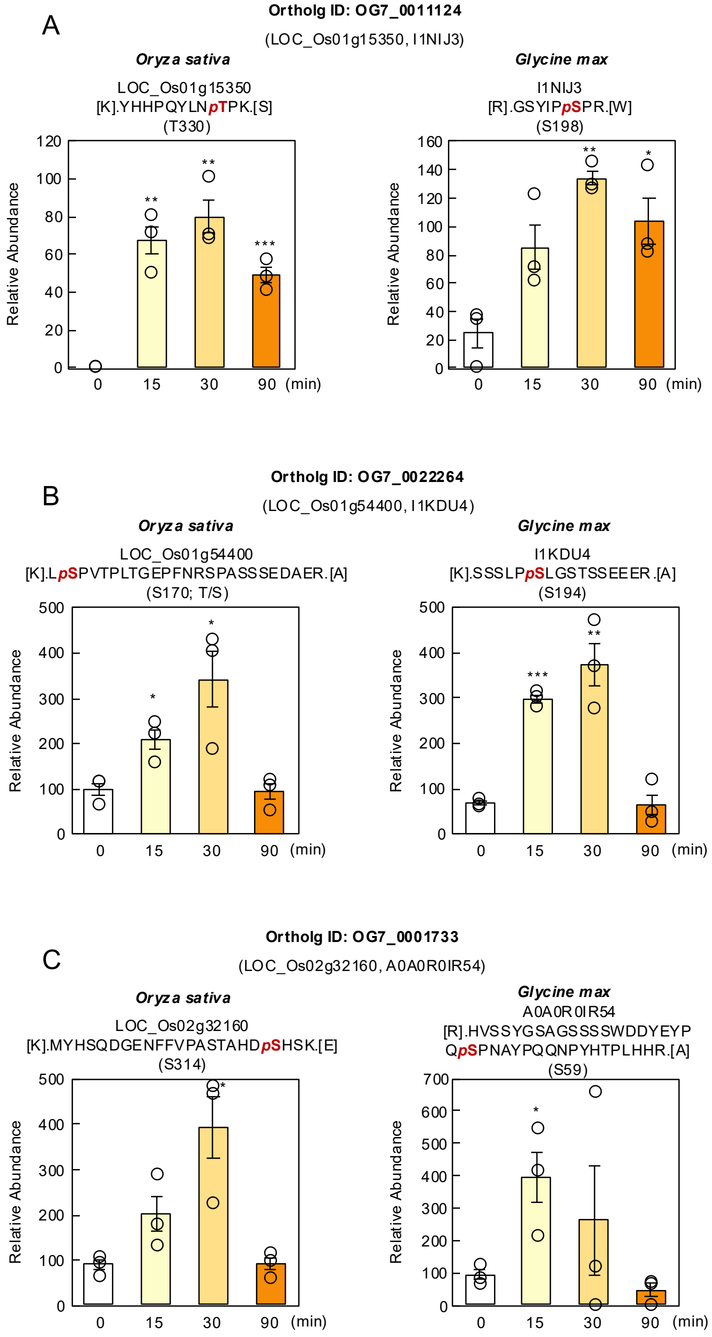

Figure.S7 ABA-responsive phosphoproteins in both rice and soybean.
(A, B and C) Relative abundance of phosphopeptides containing phosphorylated Thr330 in LOC_Os01g15350(rice), Ser198 in I1NIJ3 (soybean) (A). Relative abundance of phosphopeptides containing phosphorylated Ser170 in LOC_Os01g54400 (rice), Ser194 in I1KDU4 (soybean) (B). Relative abundance of phosphopeptides containing phosphorylated Ser314 in LOC_Os02g32160 (rice), Ser59 in A0A0R0IR54 (soybean)(C). Data are presented as mean ± SE (n = 3 biologically independent samples), and asterisks indicate significant differences as determined by Student's t-test (*P < 0.05, **P < 0.01; not significant P > 0.05).

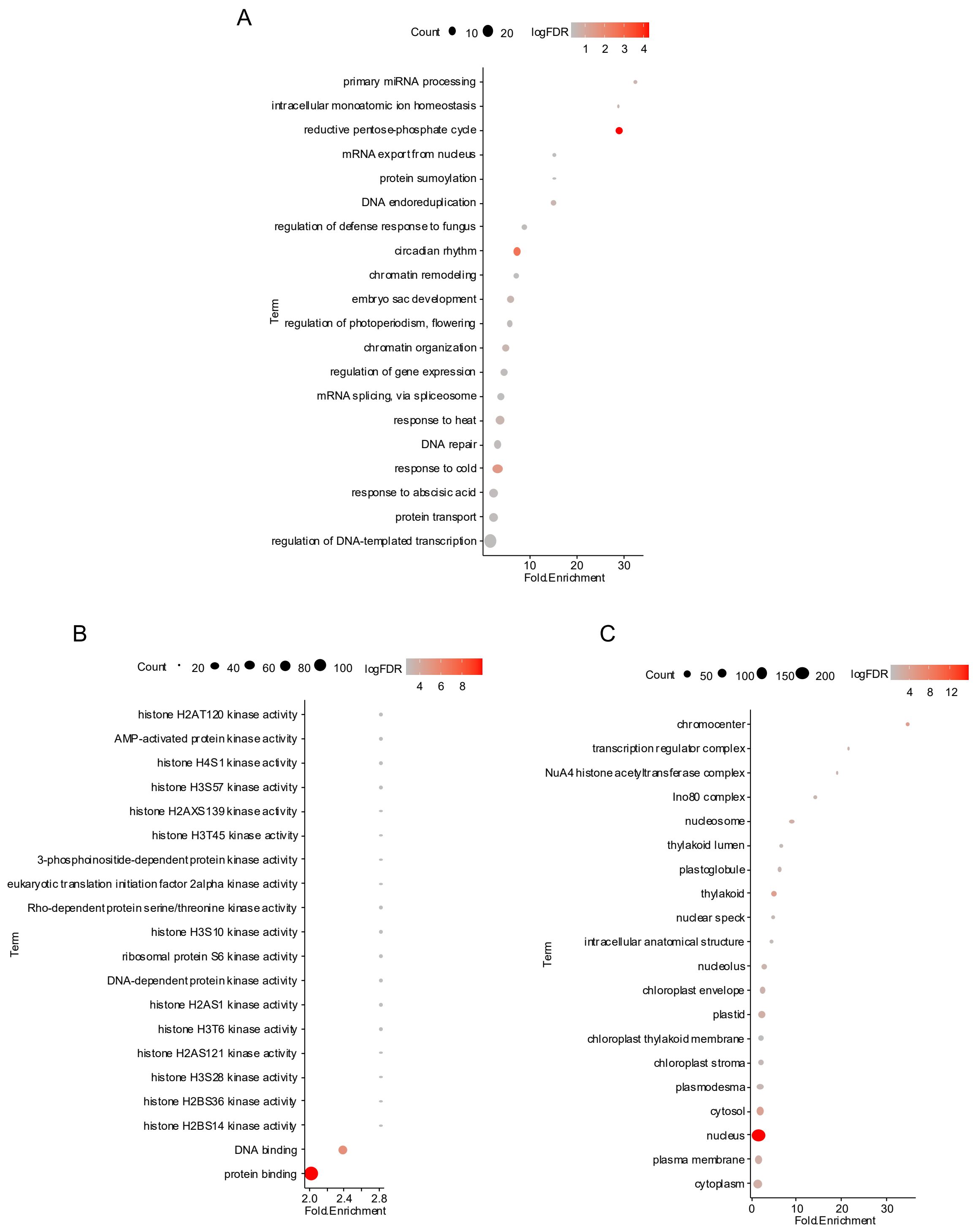

Figure.S8 GO analysis of ABA-responsive phosphoproteins in Arabidopsis root.
Arabidopsis genes included group E orthologous groups at Figure.6B were used for GO analysis. GO terms of biological process(A), molecular function(B) and cellular component(C)were evaluated by DAVID program.

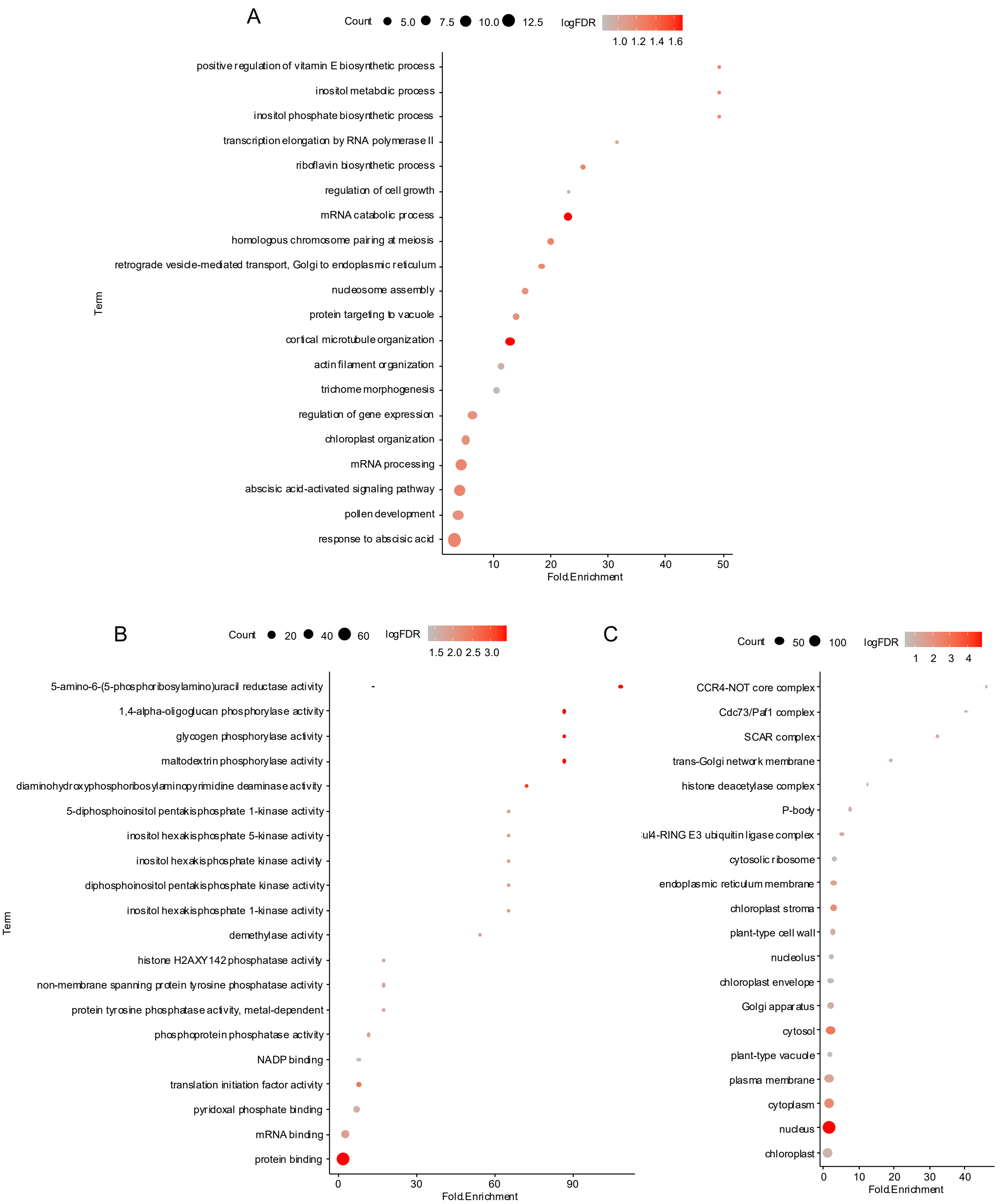

Figure.S9 GO analysis of ABA-responsive phosphoproteins in rice root.
Genes included group F orthologous groups at Figure 6B were translated representative Arabidopsis genes and used for GO analysis. GO terms of biological process(A), molecular function(B) and cellular component(C)were evaluated by DAVID program.

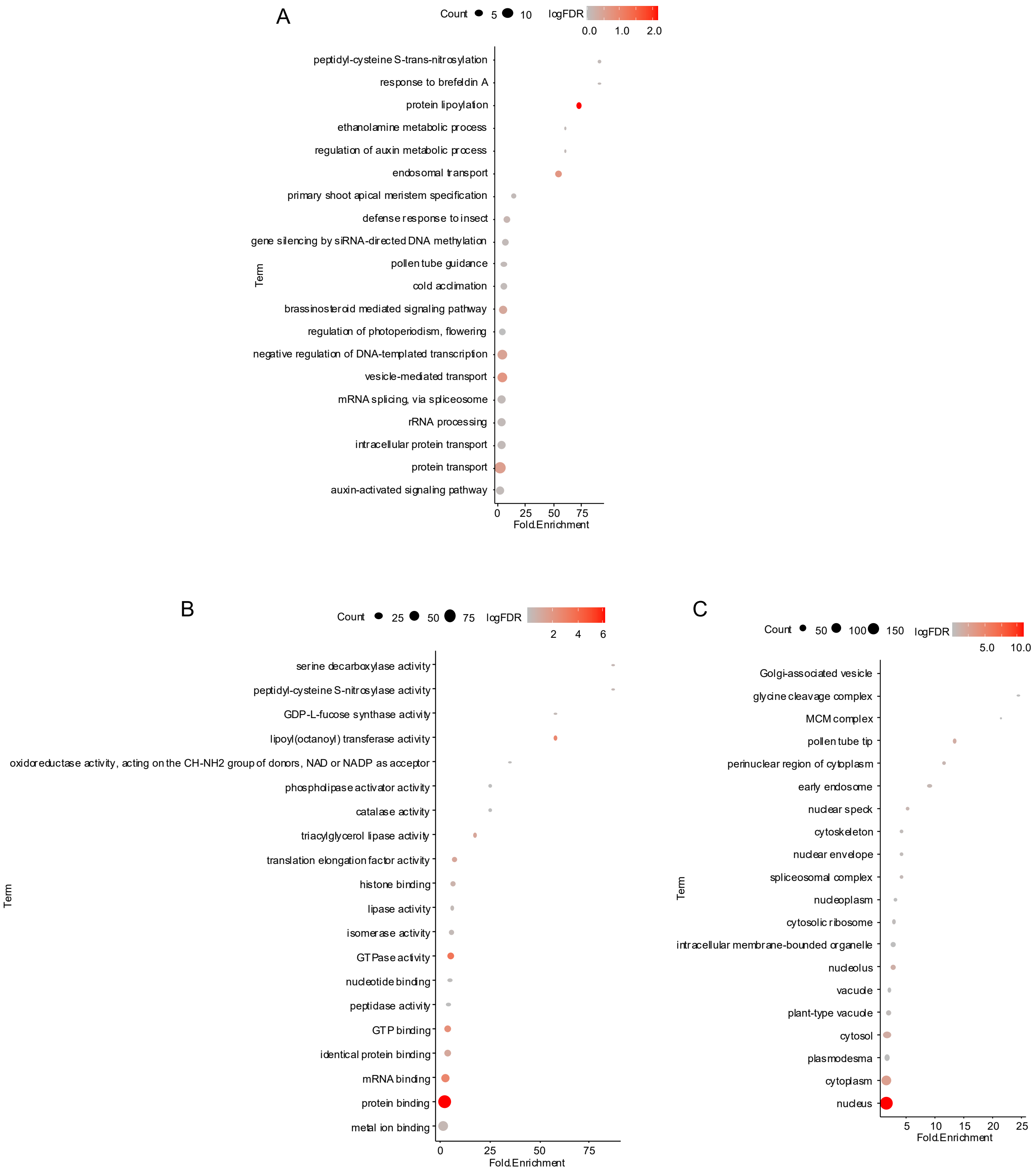

Figure.S10 GO analysis of ABA-responsive phosphoproteins in soybean root.
Genes included group G orthologous groups at Figure 6B were translated representative Arabidopsis genes and used for GO analysis. GO terms of biological process(A), molecular function(B) and cellular component(C)were evaluated by DAVID program.

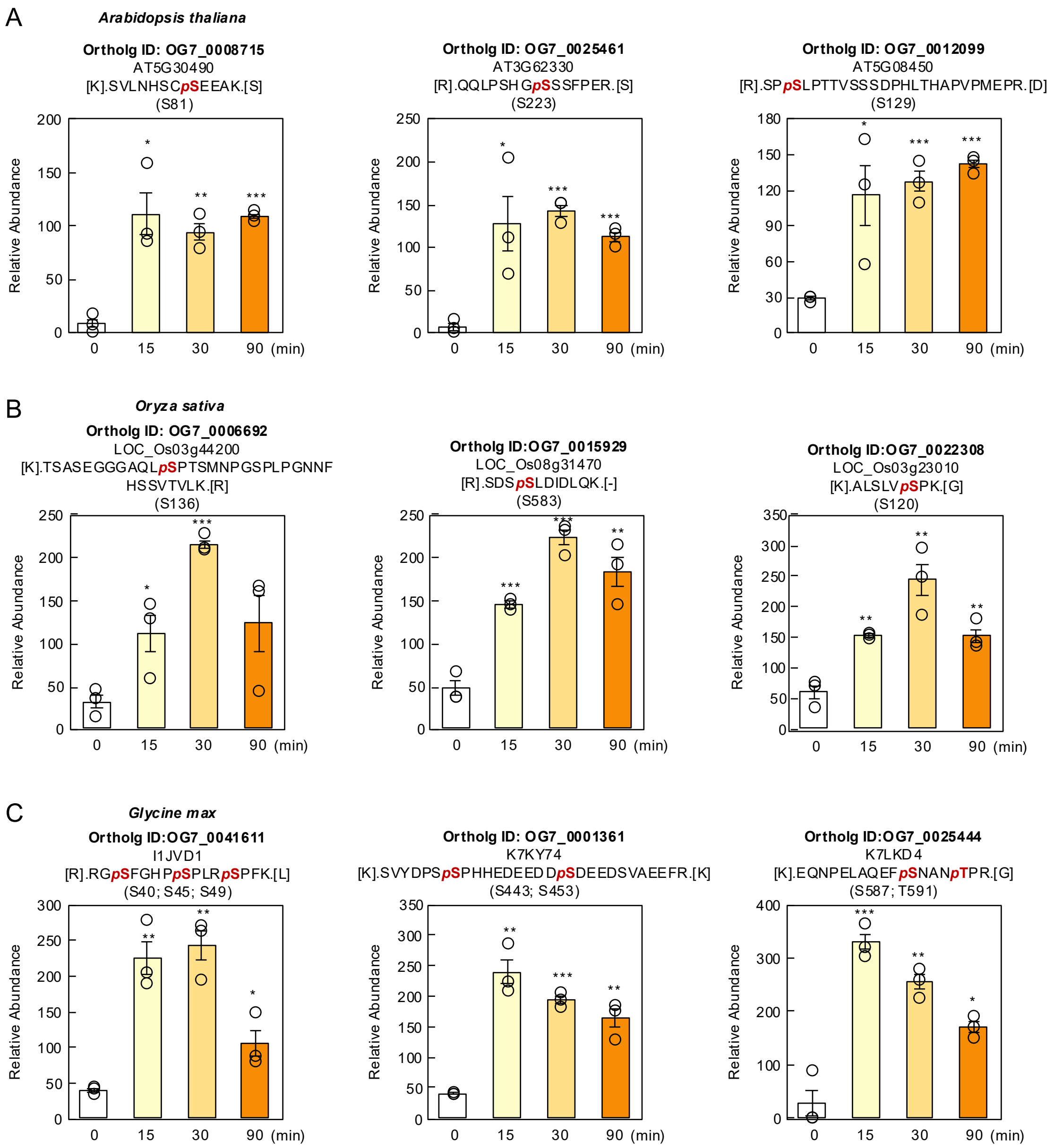

Figure.S11 ABA-responsive phosphoproteins only in Arabidopsis, rice or soybean.
(A) Relative abundance of phosphopeptides containing phosphorylated Ser81 in AT5G30490, Ser223 in AT3G62330, Ser129 in AT5G08450. (B) Relative abundance of phosphopeptides containing phosphorylated Ser136 in LOC_Os03g44200, Ser583 in LOC_Os08g31470, Ser120 in LOC_Os03g23010. (C) Relative abundance of phosphopeptides containing Ser40, Ser45 and Ser49 in I1JVD1, Ser443 and Ser453 in K7KY74, Ser587 and Thr591 in K7LKD4. Data are presented as mean ± SE (n = 3 biologically independent samples), and asterisks indicate significant differences as determined by Student's t-test (*P < 0.05, **P < 0.01; not significant P > 0.05).

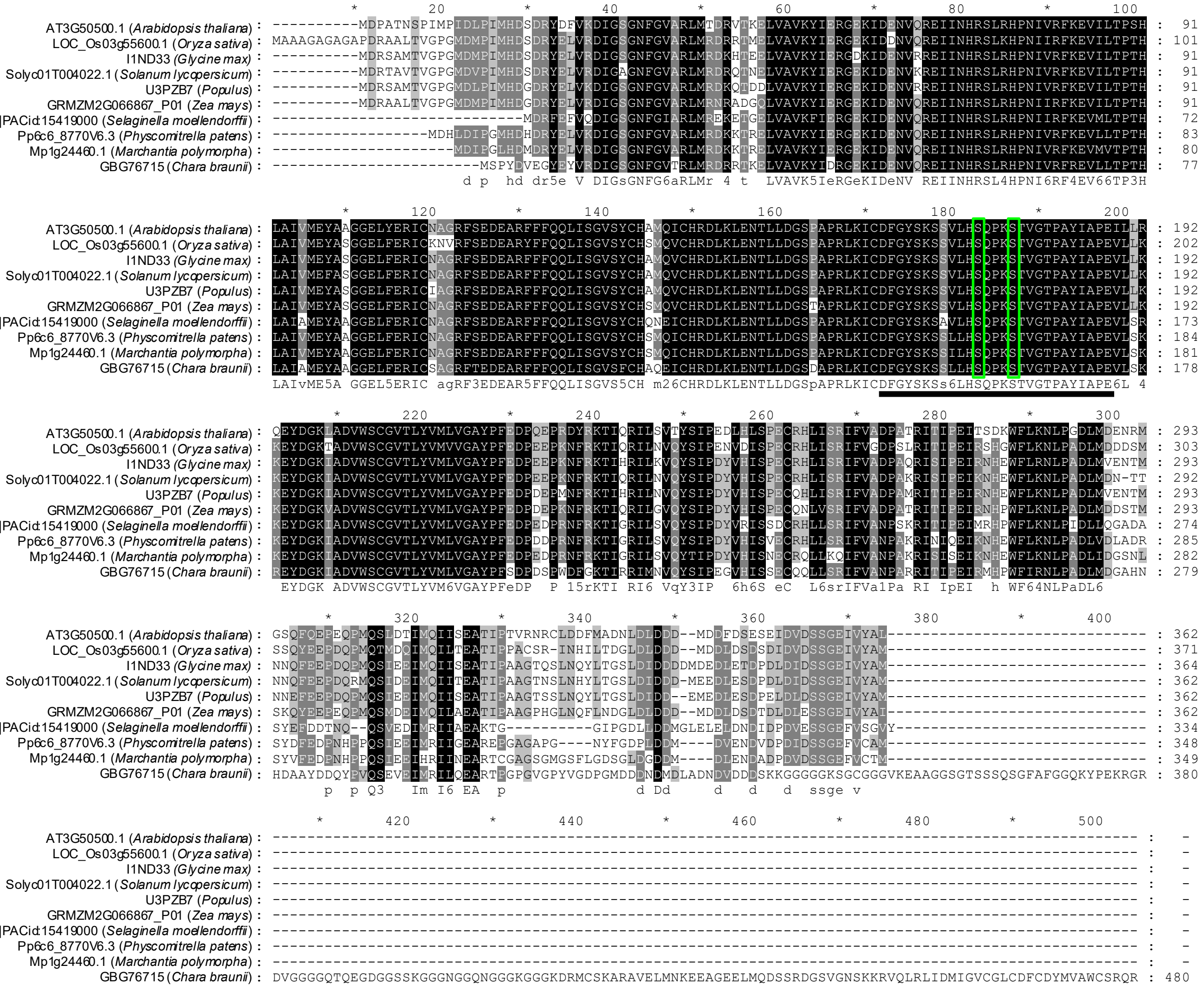

Figure.S12 Alignment of full length SnRK2s in species of Arabidopsis, rice, soybean, Solanum lycopersicum, Populus, Zea mays, Selaginella moellendorffii, Physcomitrella patens, Marchantia polymorpha, Chara braunii.

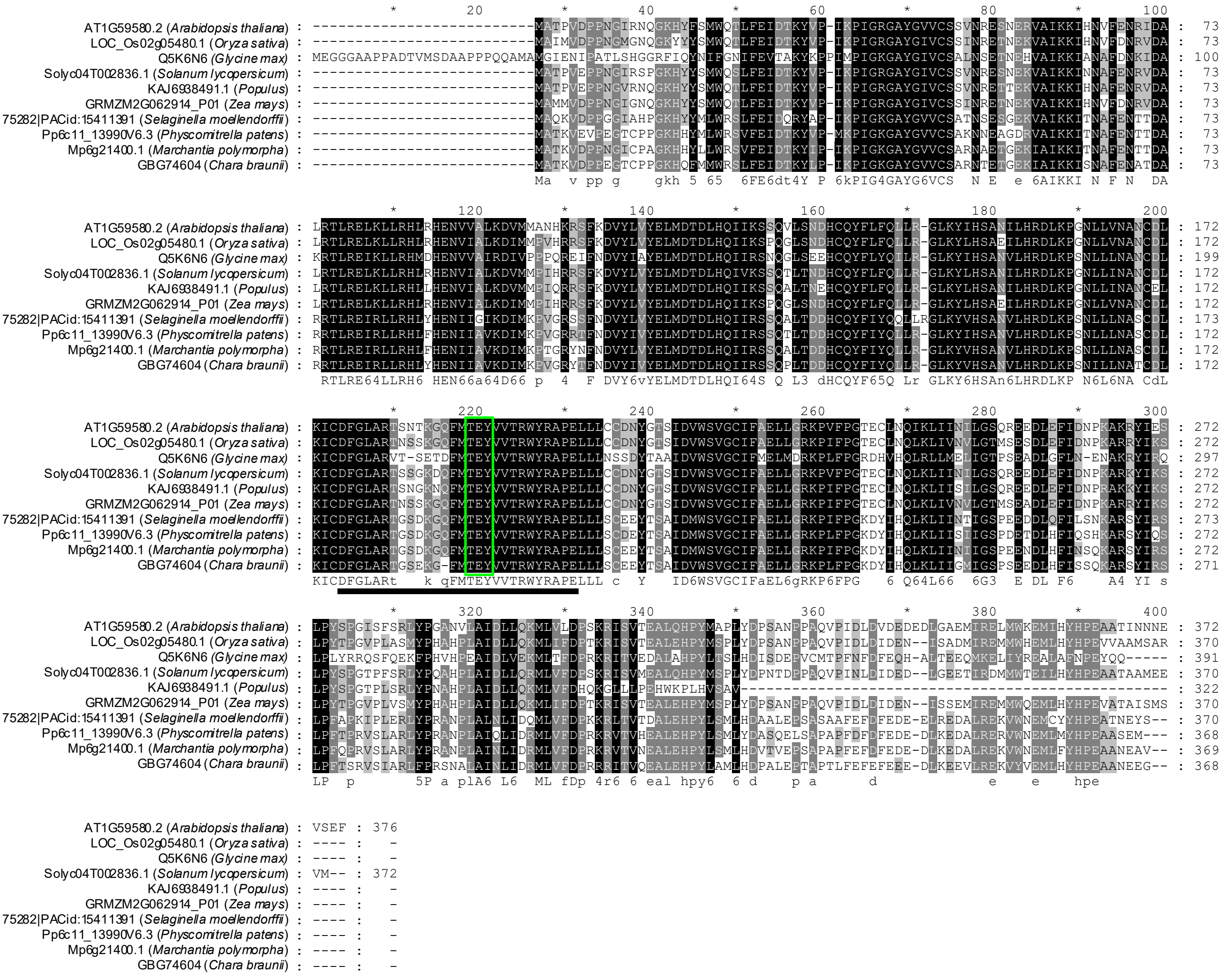

Figure.S13 Alignment of full length MAPK in species of Arabidopsis, rice, soybean, Solanum lycopersicum, Populus, Zea mays, Selaginella moellendorffii, Physcomitrella patens, Marchantia polymorpha, Chara braunii.

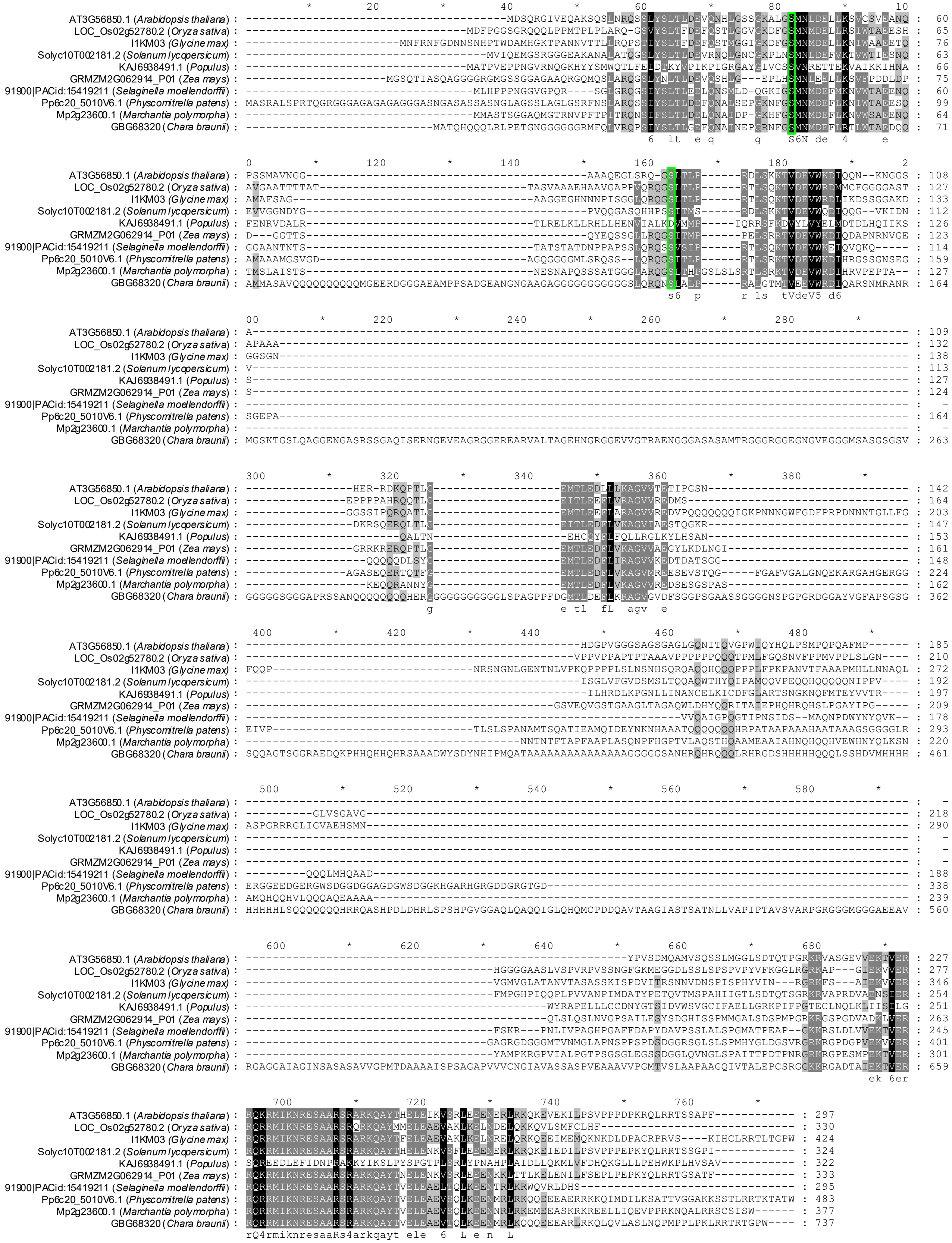

Figure.S14 Alignment of full length bZIP transcription factor in species of Arabidopsis, rice, soybean, Solanum lycopersicum, Populus, Zea mays, Selaginella moellendorffii, Physcomitrella patens, Marchantia polymorpha, Chara braunii.
